# More than 50% of *Clostridium difficile* Isolates from Pet Dogs in Flagstaff, USA, Carry Toxigenic Genotypes

**DOI:** 10.1371/journal.pone.0164504

**Published:** 2016-10-10

**Authors:** Nathan E. Stone, Lindsay C. Sidak-Loftis, Jason W. Sahl, Adam J. Vazquez, Kristin B. Wiggins, John D. Gillece, Nathan D. Hicks, James M. Schupp, Joseph D. Busch, Paul Keim, David M. Wagner

**Affiliations:** 1 Center for Microbial Genetics and Genomics, Northern Arizona University, Flagstaff, AZ, 86011, United States of America; 2 Translational Genomics Research Institute, Flagstaff, AZ, 86001, United States of America; Chang Gung University, TAIWAN

## Abstract

Nosocomial acquisition of *Clostridium difficile* is well documented, yet recent studies have highlighted the importance of community acquired infections and identified community associated reservoirs for this pathogen. Multiple studies have implicated companion pets and farm animals as possible sources of community acquired *C*. *difficile* infections in humans. To explore the potential role of pet dogs in human *C*. *difficile* infections we systematically collected canine fecal samples (*n* = 197) in Flagstaff, AZ. Additionally, nineteen fecal samples were collected at a local veterinary clinic from diarrheic dogs. We used these combined samples to investigate important questions regarding *C*. *difficile* colonization in pet canines: 1) What is the prevalence and diversity of *C*. *difficile* in this companion pet population, and 2) Do *C*. *difficile* isolates collected from canines genetically overlap with isolates that cause disease in humans? We used a two-step sequence typing approach, including multilocus sequence typing to determine the overall genetic diversity of *C*. *difficile* present in Flagstaff canines, and whole-genome sequencing to assess the fine-scale diversity patterns within identical multilocus sequence types from isolates obtained within and among multiple canine hosts. We detected *C*. *difficile* in 17% of the canine fecal samples with 10% containing toxigenic strains that are known to cause human disease. Sequencing analyses revealed similar genotypes in dogs and humans. These findings suggest that companion pets are a potential source of community acquired *C*. *difficile* infections in humans.

## Introduction

*Clostridium difficile* is an anaerobic, Gram positive, spore-forming bacillus that can colonize and proliferate in the human gut, particularly if the normal intestinal microbiota is disturbed [1]. As such, the association between the onset of *C*. *difficile* infection (CDI) following antimicrobial treatment is commonly observed [2]. *C*. *difficile* has rapidly become the most common source of antimicrobial associated diarrhea in healthcare facilities worldwide [2] and is the leading cause of hospital-associated infections in the United States [1]. An increase in the severity of patient symptoms and frequency of outbreaks was reported in hospitals from multiple countries in Europe and North America during the early 2000s, foreshadowing a continuous rise in CDI over the following decade [2]. Toxigenic strains can cause a highly variable range of symptoms, including mild diarrhea, severe pseudomembranous colitis (inflammation of epithelium infected with *C*. *difficile*), toxic megacolon (severely dilated colon), colonic perforation, and death.

The pathogenesis of *C*. *difficile* is complex, and new studies are challenging the traditional understanding of this pathogen [3, 4]. Furthermore, the presence of *C*. *difficile* spores in the gut does not always lead to colonization and CDI; some hosts appear to serve as transient carriers of the bacterium or its spores. Multiple genetically-based mechanisms are responsible for varying aspects of colonization, onset of disease, patient symptoms, and persistence of infection. *C*. *difficile* is a highly mosaic species but strains can be broadly classified as either toxigenic or non-toxigenic, with pathogenicity in humans caused by toxin-producing strains. Toxigenic *C*. *difficile* strains produce one or two glucosyltransferase exotoxins (toxins A and B), which are encoded by the *tcdA* and *tcdB* genes on the 19.6 kb pathogenicity locus (PaLoc) [3, 5]. These toxins cause apoptosis of host epithelial cells by inactivating molecules in the GTPase family (Rho, Rac, Cdc42), thus causing signaling alterations and cell death [3]. The intoxication mechanisms of toxins A and B have been the focus of much research as they are directly responsible for provoking diarrhea during CDI [3]. However, additional genetic mechanisms have been described, including mutations in the negative regulatory gene *tcdC* (also on the PaLoc), the presence of a binary toxin gene (*cdtB*), expression of other virulence factors (e.g., motility, secretion, adhesion, and immune evasion), and antimicrobial resistance; together these have been associated with increased toxicity, virulence, and disease persistence [3, 6–8]. Additionally, *C*. *difficile* has increasingly been shown to possess a highly plastic genome with a proclivity toward recombination [9], potentially leading to the emergence of novel toxin-producing genotypes that have been shown to affect both humans and animals [10].

Transmission of *C*. *difficile* is dependent on the dormant spore morphotype because vegetative cells are unable to survive the aerobic environment necessary for horizontal transmission [9]. *C*. *difficile* spores are highly resilient and are widely dispersed in the environment (possibly from transient or asymptomatically colonized hosts) and healthcare facilities [3]. The primary route of spore ingestion is contact with contaminated surfaces in the hospital setting [3] although ingestion from environmental sources is also plausible. If favorable conditions are met post-ingestion (i.e. a disrupted gut microbiota), bile salts in the small intestine induce germination and initiate the vegetative morphotype, which often leads to colonization, proliferation, and, ultimately, CDI [3]. During the course of CDI, *C*. *difficile* initiates a sporulation pathway resulting in the production of pathogenic *C*. *difficile* spores, allowing it to persist in the host and the environment and enabling horizontal transmission [9]. However, it is important to consider that the acquisition of spores does not necessarily result in disease; it is estimated that 1–3% of healthy adult humans are asymptomatically colonized [11]. Interestingly, asymptomatic colonization has been shown to be a confounding outcome that can hinder epidemiological studies because these hosts may serve as potential reservoirs for onward transmission and are not easily trackable [1, 12].

Healthcare acquisition of *C*. *difficile* is well documented, yet recent studies also have highlighted the importance of community acquired infections (CAIs) and suggested that community-associated reservoirs for this disease, such as household pets and farm animals, are likely [4, 12, 13]. An important advancement has been the application of evolutionary approaches (e.g., whole-genome sequencing) to the study of clinical cases, which enables high resolution epidemiological tracking of transmission events. One such study identified diverse sources in CDIs that were not consistent with nosocomial acquisition and concluded that community-associated transmission is more common than previously suspected [12]. It is now generally accepted that CAIs account for some unknown proportion of CDI in humans and that theses source should be explored in more detail [1, 2]. Animals have been implicated as one potential source of CAIs in humans [1, 14, 15], although the exact route of this transmission has yet to be thoroughly investigated.

The potential burden of domestic canines as a reservoir for *C*. *difficile* could be significant, as the American Veterinary Medical Association estimates dog ownership in US households to be 36.5% [16]. In this study, we hypothesized that domestic canine pets are one source of CAIs in humans. We explored this hypothesis by genotyping *C*. *difficile* obtained from canine feces in a single US city (Flagstaff, Arizona) and comparing those results to a global database of strains isolated from human CDIs (www.pubmlst.org/cdifficile) [17]. We used a two-step sequence typing approach to genotype isolates: multilocus sequence typing (MLST) to determine the broad-scale genetic diversity of *C*. *difficile* within and among Flagstaff canines, and whole-genome sequencing (WGS) to assess the fine-scale diversity among strains with identical MLST sequence types (STs) isolated from one or multiple canines. These data facilitated the investigation of three important questions regarding *C*. *difficile* in canines: 1) What is the prevalence and diversity of *C*. *difficile* in this pet population, 2) Do *C*. *difficile* isolates collected from canines genetically overlap with isolates that cause human disease, and 3) Are hypervirulent strains found in Flagstaff canines? Our findings revealed similar genotypes in dogs and humans and we demonstrate the discriminatory power of WGS to address epidemiological questions about *C*. *difficile*.

## Materials and Methods

### Sampling

**Fig 1:**
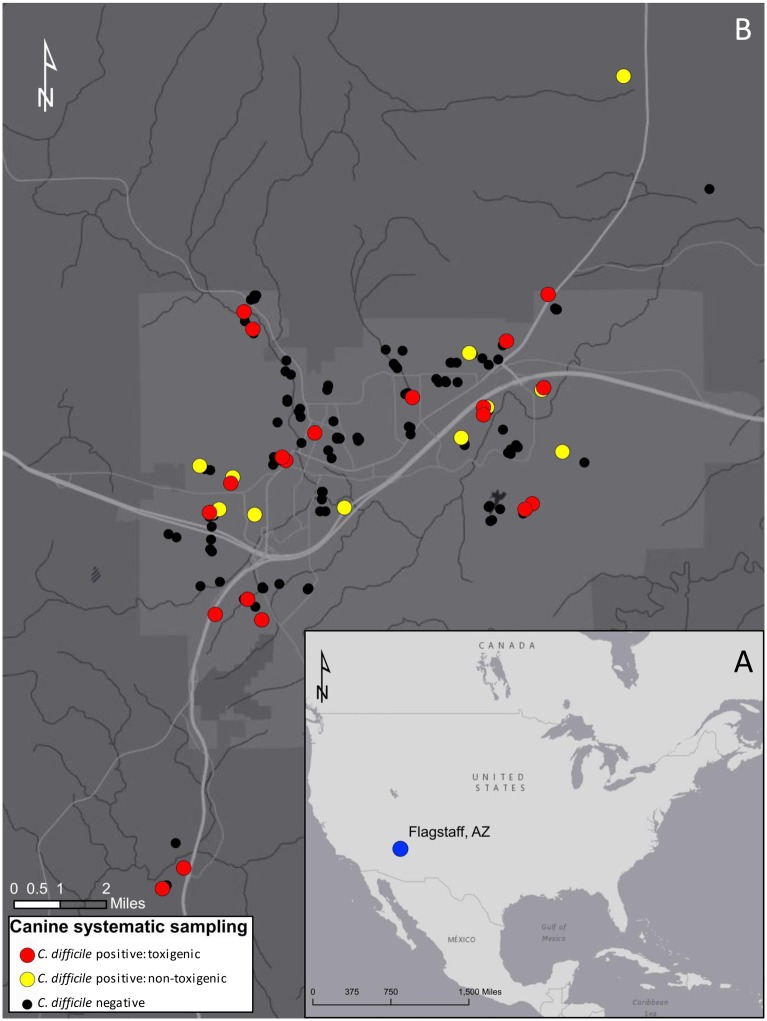
Systematically sampled canine fecal sites in Flagstaff, AZ. A) The blue dot on the inset map shows the location of Flagstaff, USA. B) *Clostridium difficile* positive fecal samples occurred throughout the sampling area (red dots represent toxigenic isolates and yellow dots represent non-toxigenic isolates), as did *C*. *difficile* negative samples (black dots). The area shaded in light grey indicates Flagstaff city limits. This map was created using ArcGIS software by Esri.

To sample the canine population of Flagstaff, Arizona, population 68,667 [18], fecal samples (*n* = 216) were collected from multiple sources. No specific permissions were required for the collection of these samples as they were obtained from either 1) public lands or 2) donated by interested third parties. We sampled from domestic canines only and none of the samples were obtained from endangered or protected species. First, we systematically collected dog fecal samples (*n* = 197) between September 22^nd^ and October 3^rd^, 2014 to represent the entire geographic range of Flagstaff, Arizona (Fig 1). Briefly, equally sized grids (*n* = 37) were overlaid on a map of all densely populated areas (locally recognized neighborhoods) within the Flagstaff city limits. Three to five fecal samples were opportunistically collected from the ground (e.g. lawns, curbsides and empty lots) within each grid in a manner to maximize the likelihood that the fecal sample came from a dog living within that grid (i.e. we avoided city parks, urban trails, and other highly frequented recreational areas where people are likely to exercise pets outside their neighborhood of origin). Most of the individual fecal samples were moderately to highly desiccated, presumably due to long periods in the environment. We collected the majority of fecal samples (*n* = 161) with this approach. Additional systematic samples originated from three sources: 1) three densely populated rural neighborhoods outside the Flagstaff city limits where pet dogs are common (*n* = 15 samples; 5 from each neighborhood), 2) interested dog owners in the Flagstaff area that voluntarily donated fecal samples (*n* = 11), and 3) two public dog parks in the city limits (*n* = 10; 5 from each park). Donated samples were only accepted if there was no *a priori* knowledge of past or present *C*. *difficile* colonization. With the exception of the 11 samples that were donated, all samples were collected anonymously. Finally, 19 canine fecal samples were collected from a local veterinary hospital between December 27^th^, 2014 and February 26^th^, 2015. These samples were obtained from diarrheic canines and were collected in accordance with Northern Arizona Universities Institutional Animal Care and Use Committee (IACUC) guidelines. All samples were stored at 4°C before processing.

### *Clostridium difficile* detection

To address the question of prevalence, we screened all fecal samples for *C*. *difficile* using a TaqMan real-time PCR assay described below (hereafter: *Cdiff* PCR). Fecal samples were enriched for *C*. *difficile* prior to extraction to increase the probability of detecting low level colonization. We use the term “colonized” only to indicate that *C*. *difficile* was present in the sample, because we cannot know with certainty which dogs were actually infected, colonized, or transient carriers of *C*. *difficile*. Approximately 1.0 gram of stool was re-suspended in 600 μL of 1x phosphate buffered saline (PBS) solution, mixed thoroughly, and heat shocked for 10 minutes at 80°C to kill any non-spore forming bacteria; this maximizes *C*. *difficile* recovery from environmental samples [19]. The suspension was then transferred to a vinyl Type C anaerobic chamber (Coy Laboratory Products, Grass Lakes, MI, USA) and split equally into two separate 2 mL tubes containing 1 mL of taurocholate-cefotoxin-cyloserine-fructose broth (TCCFB) and incubated at 36°C for 48–72 hours. The enrichments (one replicate was used for extraction, whereas the other was stored at -80°C and used for downstream culturing—see culturing section below) were then extracted using PowerSoil DNA extraction kits (MoBio, Carlsbad, CA, USA) according to manufacturers’ specifications with the following modifications: after the addition of solution C1 the samples were incubated in a hot water bath for 10 minutes at 70°C, vortexed for 30 minutes, and centrifuged at 10,000 x *g* for 30 minutes. After the addition of solutions C2 and C3 the incubation times at 4°C were increased to 1 hour and the post incubation centrifugation steps were increased to 10 minutes.

All extractions were assessed for quality and bacterial quantity using a 16S rRNA real-time PCR assay adapted from Liu *et al*. [20]. Absolute quantification was not the goal of this assay, but rather a rapid assessment of the relative bacterial DNA quantity. Therefore, we converted the previously described assay from a TaqMan to a SYBR based real-time assay by forgoing the minor groove binding (MGB) probe. PCRs were carried out in 10 μL volumes containing the following reagents (given in final concentrations): 1 μL of 1/10 diluted DNA template, 1x SYBR Green Universal master mix (Applied Biosystems, Foster City, CA, USA), 0.8 μM Betaine to increase target specificity, an additional 0.2 U Platinum *Taq* polymerase to improve efficiency (Invitrogen, Carlsbad, CA, USA), and 0.4 μM of each primer. The assay was run on an Applied Biosystems 7500 Fast Real-Time PCR System with SDS 7500 software v2.0.6 under the following conditions: 50°C for 2 minutes, 95°C for 10 minutes, and 40 cycles of 95°C for 15 seconds and 53°C for 1 minute. Positive controls and negative water controls were included on all runs. It is important to note that this 16S assay will amplify negative controls at approximately 35 CTs under these conditions due to the bacterial DNA contamination associated with the use of a cloned DNA polymerase. As such, to be conservative in our estimates of bacterial quantity, CT values greater than 30 were considered failed extractions. All PowerSoil bacterial extractions exhibited 16S amplifications between 9.82 and 21.47 CTs. As a reference, our 1ng/μL genomic DNA (gDNA) control (ATCC *C*. *difficile* strain 4118) amplified at 15.5 CT. Therefore, we concluded that the extractions were successful and should provide ample bacterial DNA to detect *C*. *difficile*, when present.

We designed a single probe TaqMan assay that is presumptively specific for *C*. *difficile*, based on an alignment of 417 publicly available *C*. *difficile* genomes (i.e. target) and 289 genomes from additional *Clostridium* species (non-target). We ran all genomes through the LS-BSR pipeline [21] to identify coding regions that are highly specific to *C*. *difficile*. Of three coding region candidates that were specific to *C*. *difficile*, we selected a 1179 bp region that encompasses an aminotransferase gene (Genbank accession # AJP12232.1). Primers and the TaqMan -MGB probe were designed using Primer Express 3.0 (Applied Biosystems, Foster City, CA, USA), where primer pair Cdiff-TaqManF (5′-GGATTGCTGATATGGATTTTAAAATACC-3′), and Cdiff-TaqManR (5′-GATACAGTTCCATAAGTTAATGTAATCCATTC-3′) generated a 189 bp PCR product and the MGB probe, Cdiff-TaqManProbe (5′-GAAGCTGTAAGAAGAGGTGTAT-3′) was fluorescently labeled with a 6FAM dye. The *Cdiff* TaqMan PCR assay was run in triplicate 10 μL reactions containing 1x TaqMan Universal PCR master mix II (w/o AmpERASE UNG) (Applied Biosystems, Foster City, CA, USA), 0.9 μM of each primer, 0.3 μM of the MGB probe, and 1 μl diluted DNA template. Additionally, this real-time PCR assay was supplemented with 0.25 U Platinum *Taq* polymerase to improve efficiency. The assay was run on an Applied Biosystems 7500 Fast Real-Time PCR System with SDS 7500 software v2.0.6 under the following conditions: 50°C for 2 minutes, 95°C for 10 minutes, and 45 cycles of 95°C for 15 seconds and 53°C for 1 minute. Positive and negative controls were included on all runs.

To ensure that any positive results from the *Cdiff* TaqMan PCR assay were indeed from *C*. *difficile*, and to confirm the specificity of this assay, we sequenced a 283 bp region that flanked the TaqMan PCR target for all positive samples. First, the fragment was amplified using forward primer Cdiff_111F (5′-TGGATTGCTGATATGGATT-3′) and reverse primer Cdiff_395R (5′-TTTGCTGATGATTCAAAGG-3′). PCRs were carried out in 10 μL volumes containing the following reagents (given in final concentrations): 2 μl diluted DNA template, 1x PCR buffer, 2.5 mM MgCl_2_, 0.2 mM dNTPs, 0.8 U Platinum *Taq* polymerase, and 0.4 μM of each primer. PCRs were thermocycled according to following conditions: 95°C for 10 minutes to release the polymerase antibody, followed by 40 cycles of 94°C for 60 seconds, 53°C for 30 seconds, and 72°C for 30 seconds. PCR products were then treated with ExoSAP-IT (Affymetrix, Santa Clara, CA, USA) using 1 μL of ExoSAP-IT per 7 μL of PCR product under the following conditions: 37°C for 15 minutes, followed by 80°C for 15 minutes. Treated products were then diluted 1/10 and sequenced in both directions using the same forward and reverse primers from the PCR in a BigDye Terminator v3.1 Ready Reaction Mix (Applied Biosystems, Foster City, CA, USA). We used 10 μL volumes for sequencing reactions containing the following reagents (given in final concentrations): 5x Sequencing Buffer, 1 μL BigDye Terminator v3.1 Ready Reaction Mix, 1 μM primer, and 5 μL diluted PCR product. The following thermocycling conditions were used: 96°C for 20 seconds, followed by 30 cycles of 96°C for 10 seconds, 50°C for 5 seconds, and 60°C for 4 minutes.

Although the possibility of false negatives exists due to low levels of *C*. *difficile* in fecal samples, we validated the sensitivity of the *Cdiff* TaqMan assay in two ways: 1) We attempted to culture *C*. *difficile* from ~10% of our TaqMan PCR negative samples (*n* = 24) (see culturing section below) and 2) we performed a serial dilution on a known concentration positive control (ATCC *C*. *difficile* strain 4118) in a complex fecal extraction background to determine the theoretical limit of detection for this assay. Briefly, we performed a serial dilution spanning eight orders of magnitude (in triplicate) ranging from 1 ng/μL to 0.1 fg/μL. These dilutions were performed using a canine fecal extraction as the solute, which was confirmed to be negative for *C*. *difficile* by *Cdiff* TaqMan PCR and culturing. Additionally, we ran a control dilution in identical fashion using molecular grade water as the solute to assess any inhibitory effects associated with a complex fecal background. No inhibitory effects were observed in the presence of a complex fecal background. The *Cdiff* TaqMan assay successfully detected *C*. *difficile* at a concentration of 1 fg, or approximately one genomic copy (based on calculations using Avogadro’s number, approximate average nucleotide weight in Daltons, and an approximate 4.3 Mb genome size: http://cels.uri.edu/gsc/cndna.html) [22]. Additionally, our attempts to culture *C*. *difficile* from 24 *Cdiff* TaqMan PCR negative samples were unsuccessful, validating the sensitivity of this assay.

To detect toxigenic *C*. *difficile* strains, a PaLoc real-time single probe detection assay (*tcdB* TaqMan PCR) was designed based on an alignment of a 7101 bp section of 503 *tcdB* (toxin B) sequences bioinformatically extracted from external genomes. To overcome nucleotide variability observed in the forward and the probe priming sites of certain strains, we designed two forward primers and two probes that were pooled together in equimolar amounts to create forward primer and probe mixes for downstream PCR. Primers and the TaqMan -MGB probes were designed using Primer Express 3.0, where primer set ToxB-TaqManF (5′- CTAGCTTATGGTCATTTGACGATGC-3′), ToxB-TaqManF2 (5′- CTAGCTTATGGTCATTTGACGATTCA -3′), and ToxB-TaqManR (5′- CTAGCTTATGGTCATTTGACGATGC-3′) generated a 176 bp PCR product and the MGB probes, ToxB-TaqManProbe (5′- TTGGTGAAGATGATAATCTTGAT-3′) and ToxB-TaqManProbe2 (5′- TTGGAGAAGATGACAATCT-3′) were fluorescently labeled with a NED dye. It is important to note that both TaqMan assays (*Cdiff* and *tcdB*) have identical reagent mix concentration and thermocycling conditions and can either be multiplexed or run independently. Positive and negative controls were included on all runs. We validated the sensitivity of the *tcdB* TaqMan real-time assay by performing a serial dilution in identical fashion to the *Cdiff* TaqMan assay described above, which yielded comparable limit of detection results.

### Anaerobic culturing of *C*. *difficile*

Culturing *C*. *difficile* from stool was performed as outlined in Edwards *et al*. [23] for all *Cdiff* TaqMan PCR and Sanger sequencing confirmed positive fecal samples (*n* = 37), except 100 μL of the previously described enrichment (see detection section above) was plated onto the taurocholate-cefotoxin-cyloserine-fructose agar (TCCFA) instead of a 1x PBS suspension. Once isolation of suspected *Clostridium* spp. was achieved, a lawn was created on brain heart infusion agar supplemented with 0.03% L-cysteine (BHIS) and incubated anaerobically at 36°C for 24–48 hours for downstream gDNA extractions. To investigate the prevalence of co-colonizations in *Cdiff* TaqMan PCR positive samples, we isolated multiple colonies from each specimen. During our preliminary research we found that co-colonizations were occasionally revealed if we isolated approximately ten colonies from *C*. *difficile* positive sample enrichments. Therefore, a goal of ten isolates from each fecal sample was established to improve the chances of detecting *C*. *difficile* co-colonizations, where present.

### *C*. *difficile* gDNA extractions and quality control

Genomic DNA was extracted from isolated colonies using DNeasy kits (Qiagen, Valencia, CA, USA) according to the manufacturer’s specifications. The gDNA was quantified on a 0.7% agarose gel using λ DNA-HindIII Digest (New England Biolabs, Ipswich, MA, USA) and diluted to 1–2 ng/μL for PCR. All isolates were screened using the previously described *Cdiff* /*tcdB* TaqMan multiplex assay to confirm them as *C*. *difficile* and to identify toxigenic strains.

Furthermore, the 16S rRNA gene was sequenced from all isolates to assess purity for downstream WGS candidates and assign species identifications to non-*C*. *difficile* isolates. The amplification and sequencing of the 16S fragment was performed as follows: First, a 466 bp fragment was amplified using the same forward and reverse primers described above from Liu *et al*. [20]. PCR reactions were carried out in 10 μL volumes containing the following reagents (given in final concentrations): 2–4 ng of DNA template, 1x PCR buffer, 2.5 mM MgCl_2_, 0.2 mM dNTPs, 0.8 U Platinum *Taq* polymerase, and 0.4 μM of each primer. PCRs were thermocycled according to following conditions: 95°C for 10 minutes to release the polymerase antibody, followed by 38 cycles of 94°C for 60 seconds, 60°C for 30 seconds, and 72°C for 30 seconds. PCR products were then treated with ExoSAP-IT using 1 μL of ExoSAP-IT per 7 μL of PCR product under the following conditions: 37°C for 15 minutes, followed by 80°C for 15 minutes. Treated products were then diluted 1/10 and sequenced in both directions using the same forward and reverse primers from the PCR in a BigDye Terminator v3.1 Ready Reaction Mix. We used 10 μL volumes for sequencing reactions containing the following reagents (given in final concentrations): 5x Sequencing Buffer, 1 μL BigDye Terminator v3.1 Ready Reaction Mix, 1 μM primer, and 5 μL diluted PCR product. The following thermocycling conditions were used: 96°C for 20 seconds, followed by 30 cycles of 96°C for 10 seconds, 50°C for 5 seconds, and 60°C for 4 minutes.

### Genotyping

Molecular typing of *C*. *difficile* has historically been performed using a variety of low-resolution genetic tools, which have proven useful for elucidating broad-scale relationships between hypervirulent and outbreak strains on a regional basis [24, 25]. However, the lack of standardization between these methods has made the global epidemiology of *C*. *difficile* difficult to investigate [1]. Gel based methods (e.g., pulse-field gel electrophoresis [PFGE], restriction endonuclease analysis [REA], and PCR-based ribotyping) are difficult to compare between laboratories because they require large reference strain collections [1]. MLST resolves this particular issue; however, other limitations do exist, namely, a single ST can correspond to multiple ribotypes or vice versa [1] and some STs occur in both toxigenic and non-toxigenic forms [26]. Nevertheless, MLST is a robust genotyping method that is highly reproducible between laboratories and provides adequate discriminatory power for generalized comparisons and the identification of co-colonizations [12, 27]. Therefore, we chose to use MLST to investigate our broad-scale diversity questions and complemented those data with WGS. The lack of a standardized typing method coupled with the complicated nature of this bacterial genome solicits the use of high resolution WGS methods to aid in the understanding of its pathology and epidemiology.

#### Multilocus sequencing typing

**Fig 2:**
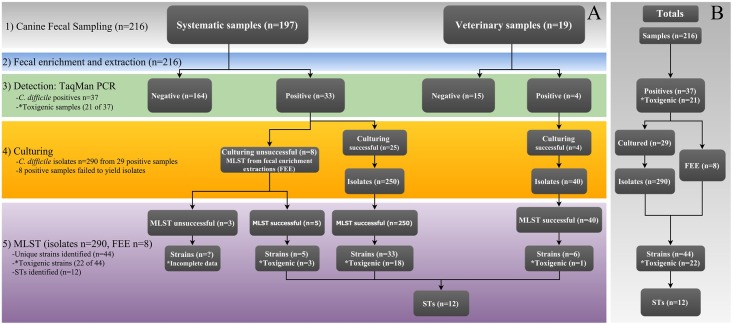
*Clostridium difficile* detection and sequence typing workflow. The prevalence and overall diversity of *C*. *difficile* in Flagstaff canines was determined by TaqMan PCR and multilocus sequence typing (MLST) [27]. A) The outcome of TaqMan PCR, culturing, and MLST from two canine sampling sources. MLST was performed on extractions from pure culture or directly from the fecal enrichment extraction (FEE), when culturing was unsuccessful (see text). B) Total outcomes from both sampling sources.

To examine genetic diversity, identify hypervirulent strains, and compare canine isolates to those collected from human CDIs, *C*. *difficile* isolates were sequence typed at multiple loci, including: 1) seven MLST loci, 2) four toxin-associated loci [27], and 3) a binary toxin gene (*cdtB*) [7]. Multiple *C*. *difficile* isolates (*n* = 1–19) were obtained from 29 of 37 *C*. *difficile* positive fecal samples (total isolates *n* = 290) (Fig 2 and S1 Table) and sequence typed using MLST as previously described [27] (S1 and S2 Tables). There were eight instances where *C*. *difficile* culture could not be obtained from a *Cdiff* PCR positive fecal sample, possibly due to non-viability of spores caused by storage conditions [28] or inconsistent shedding of this bacterium in feces, which has been observed with other bacterial species [29]. For such samples, we sequence typed directly from the fecal enrichment extraction (FEE). This approach enabled us to confirm the presence of *C*. *difficile* and identify the dominant ST in that sample enrichment, but it did not enable the detection of co-colonizations (if present). Additionally, all isolates and FEEs that were identified as toxigenic (positive for the PaLoc via *tcdB* TaqMan real-time PCR) were sequenced at *tcdA*, *tcdB*, and *tcdC* and typed as appropriate. Isolates and FEEs that were negative at *tcdB* were sequenced at *cdd1/cdu1* to confirm the absence of the PaLoc. Furthermore, all isolates and FEEs were screened for a binary toxin gene (*cdtB)* that has been suggested to be an additional virulence factor [7]. The MLST phylogenetic analysis was performed using MEGA, version 5 [30].

#### Whole-genome sequencing

To understand the fine-scale genetic differences that exist within a single ST from within and among hosts, and to evaluate the power of MLST to resolve phylogenetic relationships between strains identified in Flagstaff canines, we generated whole-genome sequences for a subset of *C*. *difficile* isolates (*n* = 54). At least one isolate from every *C*. *difficile* positive fecal sample was sequenced to investigate among host variability and, in nine instances, we sequenced multiple isolates (*n* = 2–6) with identical STs from a single fecal sample to assess within host variability (S1 and S2 Tables). In instances where co-colonizations were detected (as defined by the presence of multiple STs isolated from a single fecal sample) a representative of every unique ST was sequenced, following the methodology of Eyre *et al*. [12]. Additionally, Eyre *et al*. [12] describes a methodology for determining *C*. *difficile* transmission based on a cutoff of two core genome single-nucleotide variants (i.e. SNPs) between isolates. We used this analytical approach to determine if the fine-scale diversity identified within identical STs from within and among canine hosts was consistent with single or multiple colonizing strains.

Sequencing library preparations were performed using the same protocol as described in Keim *et al*. 2015 [31], with the following modifications for some samples. Approximately one microgram of DNA per sample was fragmented using a Q800R2 sonicator (QSonica, Newtown, CT, USA) with the following parameters: 3 minutes sonication with 15 seconds pulse on, 15 seconds pulse off, and 20% amplitude. A dual-indexing approach was adopted to reduce the amount of optical sequencing contamination, which occurs when some sequences are incorrectly assigned to samples due to sequencing errors in the indexing reads [32]. Optical contamination leads to as much as 3% of the sequences essentially crossing over from one sample into another, which cannot be bioinformatically addressed and prevents low-level detection [32]. The Illumina common primer was replaced with a second index so that both library adapters had an associated barcode. The same indices described by Kozarewa and Turner [33] were used for the second index. The samples were sequenced on an Illumina MiSeq using the 500-cycle v2 kit (Illumina, San Diego, CA, USA, Code: MS-102-2003).

**Fig 3:**
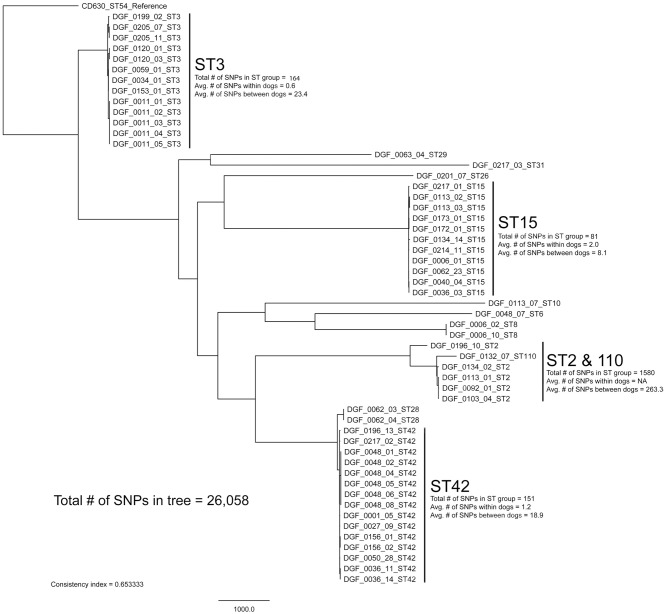
Whole-genome sequencing (WGS) maximum-likelihood phylogenetic tree containing 54 *Clostridium difficile* canine isolates from Flagstaff, USA. The total number of single nucleotide polymorphisms (SNPs) within the four most prominent sequence type (ST) groups from our study are reported. Additionally, we provided an average number of SNPs within and between canine hosts for each of these four ST groups. WGS phylogeny was rooted with reference strain CD630 [41]. The scale bar represents SNPs and the consistency index is reported.

**Fig 4:**
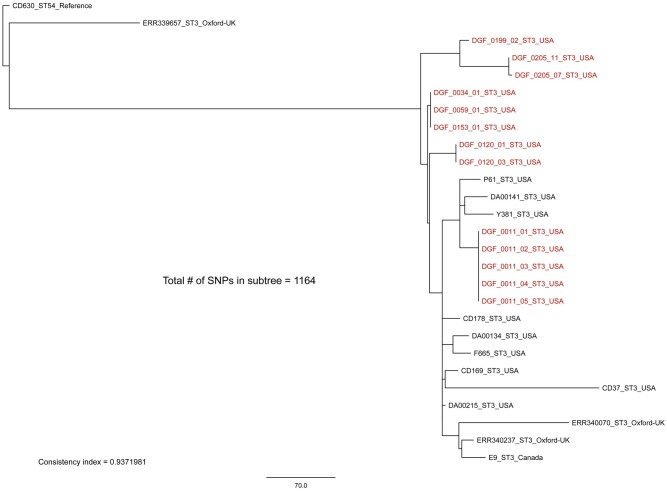
Sequence type 3 (ST3) whole-genome sequencing sub-clade tree including all ST3 isolates from this study (highlighted in red) plus publically available non-toxigenic ST3 isolates. By including global representatives of the non-toxigenic ST3 clade we see that there is geographically distinct fine-scale diversity within identical STs isolated from canines in Flagstaff, USA. Identical STs from within a single host were highly similar (0–2 SNPs), consistent with a single source of infection. The only exception to this was sample DGF_0205, which revealed 12 SNPs between isolates. This result is indicative of multiple colonizing strains that happened to be of the same ST (see text). The maximum-likelihood phylogeny was rooted with reference strain CD630 [41]. The scale bar represents SNPs and the consistency index is reported.

**Fig 5:**
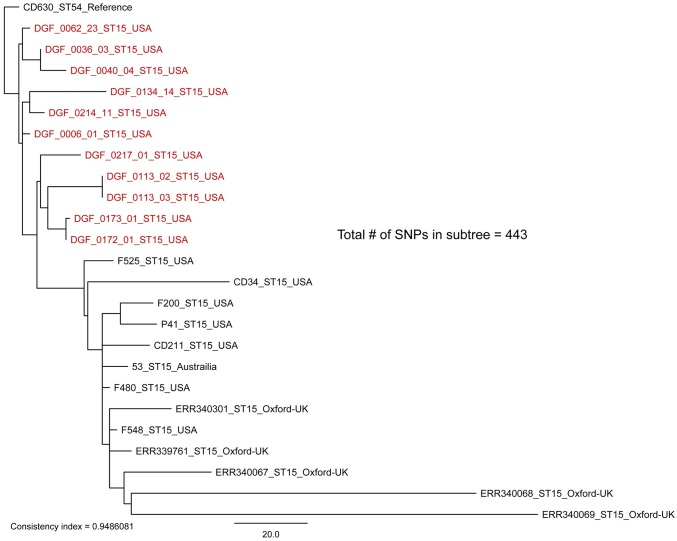
Sequence type 15 (ST15) whole-genome sequencing sub-clade tree including all ST15 isolates from this study (highlighted in red) plus publically available ST15 isolates. By including global representatives of ST15 we see that there is geographically distinct fine-scale diversity within identical STs isolated from canines in Flagstaff, USA. Identical STs from within a single host were highly similar (0–2 SNPs), consistent with a single source of infection. The maximum-likelihood phylogeny was rooted with reference strain CD630 [41]. The scale bar represents SNPs and the consistency index is reported.

**Fig 6:**
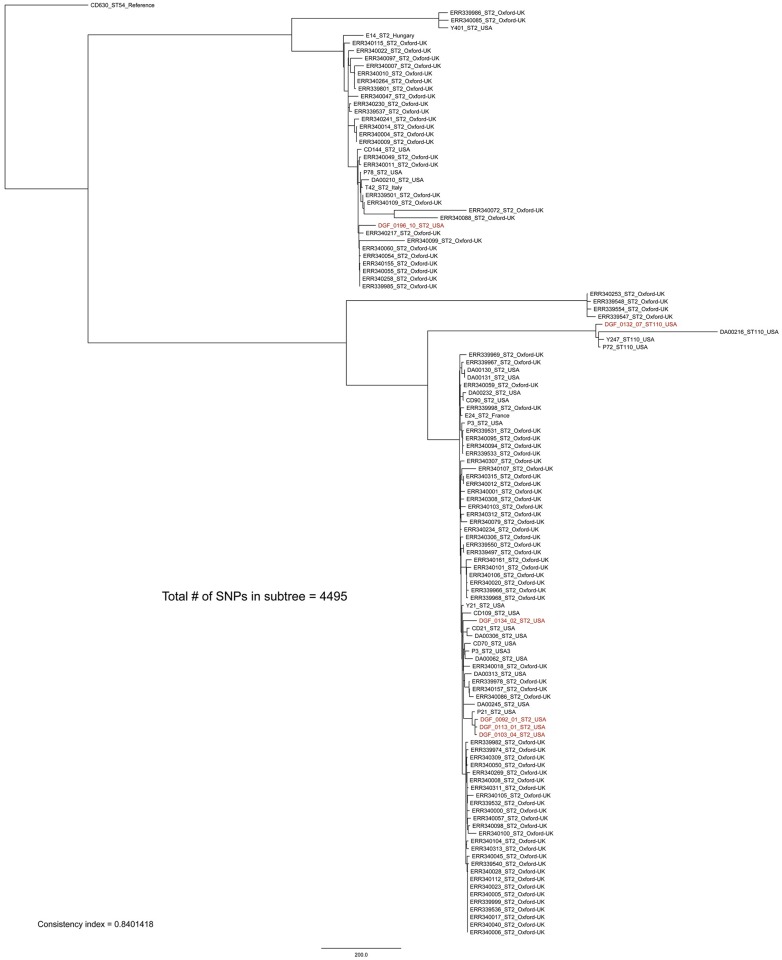
Sequence type 2 (ST2) and 110 whole-genome sequencing sub-clade tree including all ST2 and 110 isolates from this study (highlighted in red) plus publically available ST2 and 110 isolates. By including global representatives of this ST group we see that much of the global fine-scale diversity within these STs (1580/4495 SNPs or 35%) was represented in isolates from canines in Flagstaff, USA. The maximum-likelihood phylogeny was rooted with reference strain CD630 [41]. The scale bar represents SNPs and the consistency index is reported.

**Fig 7:**
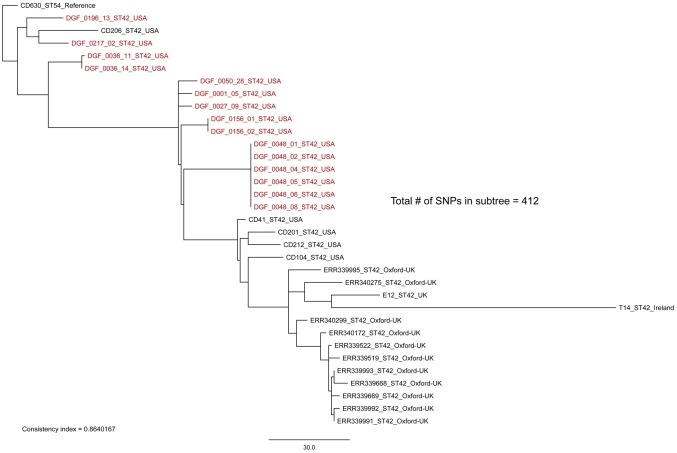
Sequence type 42 (ST42) whole-genome sequencing sub-clade tree including all ST42 isolates from this study (highlighted in red) plus publically available ST42 isolates. By including global representatives of ST42 we see that there is geographically distinct fine-scale diversity within identical STs isolated from canines in Flagstaff, USA. Identical STs from within a single host were highly similar (0–2 SNPs), consistent with a single source of infection. The maximum-likelihood phylogeny was rooted with reference strain CD630 [41]. The scale bar represents SNPs and the consistency index is reported.

All sequenced genomes were aligned against the finished *C*. *difficile* genome, CD630 (Genbank accession # AM180355.1) with BWA-MEM [34]. Single nucleotide polymorphisms (SNPs) were identified with the UnifiedGenotyper method in GATK v2.5.2 [35, 36]. Single nucleotide polymorphisms that fell within duplicated regions, as identified by a self-alignment of the reference with NUCmer [37], were filtered from downstream analyses. All of these methods were wrapped by the Northern Arizona SNP Pipeline (http://tgennorth.github.io/NASP/) [38]. A maximum-likelihood phylogeny was inferred on the concatenated SNP alignment (*n* = 55018 SNPs) with RAxML v8 [39], using the ASC_GTRGAMMA substitution model (general time-reversible model with a gamma-distributed rate of substitutions that takes into account ascertainment bias) with the Lewis correction. The global phylogeny (Fig 3) was inferred on a core genome SNP alignment that contained clean SNP calls for all genomes at all sites. For the sub-clade tree comparisons (Figs 4–7), genomes were parsed from a matrix that contains all called SNPs, and a new SNP alignment was generated for core genome SNPs for that set of genomes. The consistency index for each SNP alignment was calculated with Phangorn [40]. The pairwise number of SNPs between samples was identified from the NASP matrix without correction.

#### Detection of antimicrobial resistance determinants

We bioinformatically screened all WGS isolates for five common antimicrobial resistance determinants (S1 and S2 Tables) [8]. Using publically available sequences from PubMLST (www.pubmlst.org/cdifficile) [17], we queried our WGS assemblies with LS-BSR for the presence of the *tet*(M) gene on the Tn5397 transposon that is associated with tetracycline and chloramphenicol resistance (Genbank locus tag: HMPREF0868_1202), and the *erm*(B) gene (Genbank locus tag: EGYY_26800) on the Tn5398 transposon that is associated with erythromycin resistance; a BSR [42] value >0.8 was assumed to be conserved (i.e. gene present). From the NASP matrix, we also queried one *gyrA* and four *gyrB* gene amino acid substitutions associated with resistance to fluoroquinolones, as well as five *rpoB* gene amino acid substitutions associated with rifampicin resistance [8].

### Data sharing

All WGS reads generated during this study have been deposited in NCBI BioProject database under accession # PRJNA309189. The associated SRA numbers for these 54 isolates are sequentially assigned beginning with # SRR3115454 and ending with # SRR3115507 (S1 Table). Multilocus sequence types for one representative of the 44 unique isolates discovered during this study have been deposited in the isolate database of www.pubmlst.org/cdifficile [17].

## Results and Discussion

The role of canines as a possible source of human *C*. *difficile* CAIs has not been explored in great detail. Recent investigations have used PCR ribotyping to infer the potential for transmission between dogs and humans [43–47], but evidence using high-resolution genotyping methods has yet to be presented. The US is a pet friendly country with dog ownership estimated at 36.5% of households [16], which is nearly double the rate from any other country that reports these statistics [48]. Furthermore, *C*. *difficile* carriage rates in healthy dogs have been previously estimated in the range of 0–10% [49]. Together, these reports suggest that the role of canines as a reservoir for *C*. *difficile* and a subsequent source of human CAIs could be significant, particularly in the US.

*C*. *difficile* was widely dispersed in Flagstaff canines (i.e. no spatial clusters were identified); highlighting the potential that pet canines throughout Flagstaff could serve as a *C*. *difficile* reservoir (Fig 1). The overall colonization rate was 17.1% (37/216) in the canine fecal samples, of which 56.8% (21/37; 9.72% of overall total) contained toxigenic STs (Fig 2). However, none of the ten samples collected at two dog parks were positive for *C*. *difficile*. Interestingly, the rate of detection was relatively equivalent between the systematically collected samples and those obtained from the veterinary clinic, 16.8% (33/197) versus 21.1% (4/19), respectively (Fig 2). At the onset of this study we expected to find a higher prevalence of toxigenic strains from the veterinary samples because we presumed *C*. *difficile* to be an occasional cause of diarrhea in canines. However, only one toxigenic strain was identified from the six veterinary samples. Conversely, 55.3% (21/38) of the strains from the systematically collected samples were predicted to be toxigenic by *tcdB* TaqMan PCR (Fig 2). In addition, there was no observed association between the softness of the canine stool samples and the detection of toxigenic *C*. *difficile*, regardless of the sampling source (data not shown). These observations are consistent with asymptomatic carriage of toxigenic *C*. *difficile* in canines, as has been suggested in other studies that found it was frequently isolated from both clinically normal and diarrheic dogs [44, 49].

**Fig 8:**
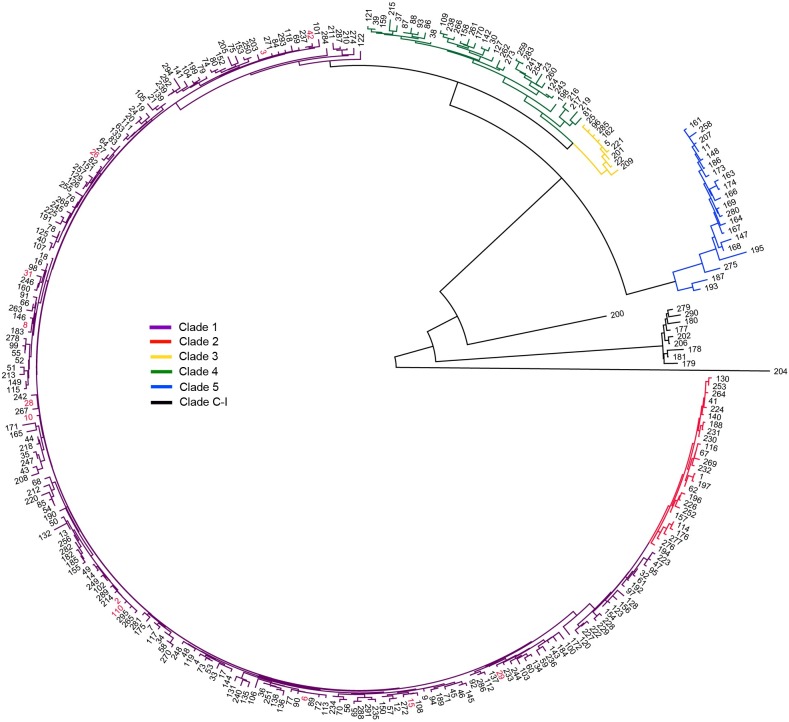
Multilocus sequence typing (MLST) neighbor-joining tree. This tree was constructed using the concatenated sequences of all available *Clostridium difficile* sequence types (STs) from www.pubmlst.org/cdifficile with the 12 STs from clade 1 found in Flagstaff canines highlighted in red font [17]. No other clade was represented by the STs identified in this study. The tree was rooted using ST204 from clade C-I. Clade descriptions are the same as previously described [26].

Our findings provide new insights into the diversity of *C*. *difficile* in canines and reveal that similar genotypes are present in both dogs and humans. We identified 44 unique strains within the 37 positive canine fecal samples (S1 and S2 Tables, and Fig 2), representing twelve distinct STs (Table 1 and Fig 2). Although all twelve of the canine STs fell into the genetically diverse and non-hypervirulent clade 1 of the MLST global phylogeny (Fig 8) [26, 27], 11 have been isolated from humans and six are among the most frequently identified in human CDIs (2, 8, 42, 6, 3, and 10) (Table 1) [6]. It is important to point out that only non-toxigenic forms of ST3 and ST31 were detected from canine samples in this survey, whereas strains of these STs isolated from human CDIs in other studies were toxigenic (Table 1). All *C*. *difficile* in our study (*n* = 290 isolates and 8 FEEs) were negative for an important binary toxin gene (*cdtB*) that has been associated with increased virulence among certain strains, but is not associated with colonization efficacy or disease outcome in humans [50–52]. Additionally, we identified four (out of 54) WGS isolates that carried antimicrobial resistance determinants (S1 and S2 Tables). These findings may suggest that canines could serve as a potential source of community acquired non-hypervirulent *C*. *difficile* in humans, and canine strains could lead to antimicrobial-induced disease in the clinical setting. We propose this as a hypothesis that requires further exploration.

**Table 1:** All sequence types (STs) identified in this study and their association with human disease.

ST	Number of canine fecal samples that carried ST	Toxigenic ST?	Isolated from humans? (*n* patients)*
2	7	Yes	Yes (104)
8	1	Yes	Yes (96)
42	10	Yes	Yes (72)
6	1	Yes	Yes (67)
3	8	Yes/No†	Yes (59)
10	1	Yes	Yes (47)
15	11	No	Yes (8)
28	1	Yes	Yes (2)
26	1	No	Yes (1)
31	1	Yes/No†	Yes (1)
110	1	Yes	Yes (1)
29	1	No	No

Eleven of the 12 STs have been isolated from human *Clostridium difficile* infections with six being among the most commonly isolated non-hypervirulent STs (2, 8, 42, 6, 3, and 10) [6].

*Data retrieved from www.pubmlst.org/cdifficile [17]

^†^Isolates from humans were toxigenic, fecal samples were not

Compared to the reported rate of human co-infections (<10%) [53], we identified a striking level of *C*. *difficile* diversity within single canine hosts. By examining multiple isolates (*n* = 2–19) from 28 of the *Cdiff* PCR positive fecal samples, we uncovered eight instances (28.6%) of *C*. *difficile* co-colonization (i.e. the presence of multiple STs from a single host) (Table 2), which suggests that the isolation of multiple colonies from a sample (~10 in this study) is essential for characterizing the population of genotypes that may be present in a single host. Co-infection increases the risk of bringing together toxigenic strains with antimicrobial-resistant ones. For example, we found one Flagstaff dog that carried both a non-toxigenic isolate (ST15) with the *erm(B)* antimicrobial resistant transposon and a toxigenic strain (ST2) for which no antimicrobial-resistance determinants could be identified (see sample DGF_0134 in S1 and S2 Tables). A similar combination was also found in sample DGF_0196 from our study (S1 and S2 Tables). If horizontal gene transfer were to occur, the resulting strains would become a greater problem for public health. Indeed, horizontal gene transfer has been suggested to be responsible for the dissemination of antimicrobial-resistance determinants in a wide variety of diverse bacterial species [54]. Furthermore, the possibility of co-infection has been used as a presumptive explanation for seemingly unlinked epidemiological cases [12], since under-sampling the diversity within a single host may cause linked cases to be overlooked.

**Table 2:** *Clostridium difficile* co-colonizations observed in canines.

Sample ID	# of Isolates†	Sequence Type							
		2*	6*	8*	10*	15	28*	31	42*
DGF_0006	11			+		+			
DGF_0036	15					+			+
DGF_0048	13		+						+
DGF_0062	17					+	+		
DGF_0113	10	+			+	+			
DGF_0134	15	+				+			
DGF_0196	19	+							+
DGF_0217	8					+		+	+

We identified eight fecal samples that carried multiple *C*. *difficile* sequence types (STs). The non-toxigenic ST15 was the most commonly identified ST in these co-colonizations.

*Toxigenic sequence type

^†^For co-colonization abundance details see S1 and S2 Tables

Our WGS phylogenetic analysis of identical STs within and among canine hosts revealed additional SNP diversity, similar to what has been observed in humans [12], and suggests that future studies testing the hypothesis that canines serve as a source of human CDIs should utilize WGS-based analyses. With one exception, replicate isolates of the same ST from single hosts differed on average by ≤2 WGS SNPs, qualifying them as epidemiologically linked. The exception was between isolates DGF_0205_07 and DGF_0205_11, which differed by 12 SNPs (Fig 4). Since recombination is fairly common in *C*. *difficile* [13], it is possible for multiple SNPs to transfer as the result of a single mutational event. Therefore, we performed an analysis of SNP density to investigate the proximity of these 12 SNPs. This analysis suggested that these 12 SNPs were likely not the result of a recombination event(s) as they were distributed throughout the core genome and not clustered within a single gene or gene cassette (data not shown). As such, we infer that this host was colonized with multiple distinct strains that just happened to be of the same ST. This demonstrates that sequencing only one strain of each unique ST from each positive sample was appropriate in most circumstances (8 of 9), but there is still the possibility of underestimating within host diversity when using this methodology. Since the presence of multiple colonizing strains of the same ST was the most likely explanation for these 12 SNPs, we chose not to include this comparison in the calculation of the average number of SNPs within single samples reported in Fig 3. In contrast to these patterns from single hosts, identical STs from different dogs were inconsistent with a recent transmission event, differing by >10 WGS SNPs on average (Figs 3–7) [12].

The spore morphotype is important for dog-mediated dispersal and plausible horizontal transmission into humans because of its ability to persist for long periods under aerobic conditions [9]. An important consideration for the development and dispersal of *C*. *difficile* spores is the length of time that a host is colonized. For instance, asymptomatic colonization may be associated with extended periods of survival of vegetative cells in the intestinal tract, which could facilitate an optimal sporulation pathway due to the nutrient starvation that naturally occurs via passage though the gut of a healthy host [3, 9]. If this is true, then asymptomatic carriers could potentially shed spores more consistently than symptomatic hosts (albeit in lower abundance) because acutely ill hosts tend to shed vegetative cells rapidly, before the development of spores can take place. Furthermore, the *C*. *difficile* cells in a symptomatic host are more likely to experience medical interventions geared toward disease eradication. As such, asymptomatic canine carriers of *C*. *difficile* may serve as long term reservoirs and play an important role in the epidemiology of this pathogen.

## Conclusions

Canines in Flagstaff, USA carry diverse *C*. *difficile* strains with STs from clade 1 that are known to cause human disease. This study provides a snapshot in time of the prevalence, diversity, and complexity of *C*. *difficile* colonization in this canine population and supports two important findings; 1) *C*. *difficile* types similar to those that cause disease in humans are widespread in dog feces and 2) Canines could serve as a source of community acquired CDIs in humans. Whole-genome sequencing is emerging as an integral tool for studying the epidemiology of *C*. *difficile*, which traditionally has relied on gel-based typing methods that are difficult to standardize across laboratories and provide limited information. The use of evolutionary analyses facilitated by WGS is illuminating novel and important insights into this global pathogen [12, 15]. Areas for future research include investigating human derived *C*. *difficile* isolates from symptomatic and healthy adults, as well as paired samples from humans and dogs within a single household.

## Supporting Information

S1 Table All isolates (*n* = 290) and fecal enrichment extractions (*n* = 8) with sampling source, sequence type, toxin genotype, and antimicrobial resistance determinant status. (DOCX)

S2 Table Summarized Table S1. (DOCX)
