# Supplementary material for: More than 50% of Clostridium difficile Isolates from Pet Dogs in Flagstaff, USA, Carry Toxigenic Genotypes
Source: PLoS One. 2016 Oct 10;11(10):e0164504. doi: 10.1371/journal.pone.0164504 (PMC5056695; doi:10.1371/journal.pone.0164504)
Supplement: S1 Table — (DOCX) [file pone.0164504.s001.docx]

**Supplemental Files: Domestic Canines are a Potential Source of Community Acquired *Clostridium difficile* Infections in Humans**

**Table S1. All isolates (*n*=290) and fecal enrichment extractions (*n*=8) with sampling source, sequence type, toxin genotype, and antimicrobial resistance determinant status.**

| Sample ID | | | Isolate ID | | Source | | *Cdiff* TaqMan® | *tcdB* TaqMan® | MLST | | Toxin genes | | | | | | | WGS | Resistance determinants | | | | | NCBI SRA # |
| --- | --- | --- | --- | --- | --- | --- | --- | --- | --- | --- | --- | --- | --- | --- | --- | --- | --- | --- | --- | --- | --- | --- | --- | --- |
|  |  |  |  |  |  |  |  |  | ST | Clade | *tcdA* | *tcdB* | *tcdC*^‡^ | *tcdC*Δ | *cdd1*/*cdu1* | *cdtB* toxin | Toxin genotype |  | *tet(M)* | *erm(B)* | *gyrA* | *gyrB* | *rpoB* |  |
| Cdiff-ATCC | | | ATCC_4118 | | Control | | + | + | 1 | 2 | + | 8 | 1 | Δ1:Δ18 | - | + | A^+^B^+^C^Δ1Δ18^*cdtB*^+^ | x | - | - | Thr-82→Ile | - | - | NA |
| Cdiff-ATCC | | | ATCC-4118 | | Control | | + | + | 1 | 2 | + | 8 | 1 | Δ1:Δ18 | - | + | A^+^B^+^C^Δ1Δ18^*cdtB*^+^ |  | NA | | | | | |
| DGF_0001 | | | DGF_0001_04 | | Systematic | | + | + | 42 | 1 | + | 3 | 3 | WT | - | - | A^+^B^+^C^WT^*cdtB*^-^ |  |  |  |  |  |  |  |
| DGF_0001 | | | DGF_0001_05 | | Systematic | | + | + | 42 | 1 | + | 3 | 3 | WT | - | - | A^+^B^+^C^WT^*cdtB*^-^ | x | - | - | - | - | - | SRR3115454 |
| DGF_0006 | | | DGF_0006_01 | | Systematic | | + | - | 15 | 1 | - | - | - | - | + | - | absent | x | - | - | - | - | - | SRR3115455 |
| DGF_0006 | | | DGF_0006_02 | | Systematic | | + | + | 8 | 1 | + | 3 | 3 | WT | - | - | A^+^B^+^C^WT^*cdtB*^-^ | x | - | - | - | - | - | SRR3115456 |
| DGF_0006 | | | DGF_0006_03 | | Systematic | | + | + | 8 | 1 | + | 3 | 3 | WT | - | - | A^+^B^+^C^WT^*cdtB*^-^ |  | NA | | | | | |
| DGF_0006 | | | DGF_0006_04 | | Systematic | | + | + | 8 | 1 | + | 3 | 3 | WT | - | - | A^+^B^+^C^WT^*cdtB*^-^ |  |  |  |  |  |  |  |
| DGF_0006 | | | DGF_0006_05 | | Systematic | | + | + | 8 | 1 | + | 3 | 3 | WT | - | - | A^+^B^+^C^WT^*cdtB*^-^ |  |  |  |  |  |  |  |
| DGF_0006 | | | DGF_0006_06 | | Systematic | | + | + | 8 | 1 | + | 3 | 3 | WT | - | - | A^+^B^+^C^WT^*cdtB*^-^ |  |  |  |  |  |  |  |
| DGF_0006 | | | DGF_0006_07 | | Systematic | | + | + | 8 | 1 | + | 3 | 3 | WT | - | - | A^+^B^+^C^WT^*cdtB*^-^ |  |  |  |  |  |  |  |
| DGF_0006 | | | DGF_0006_08 | | Systematic | | + | + | 8 | 1 | + | 3 | 3 | WT | - | - | A^+^B^+^C^WT^*cdtB*^-^ |  |  |  |  |  |  |  |
| DGF_0006 | | | DGF_0006_09 | | Systematic | | + | + | 8 | 1 | + | 3 | 3 | WT | - | - | A^+^B^+^C^WT^*cdtB*^-^ |  |  |  |  |  |  |  |
| DGF_0006 | | | DGF_0006_10 | | Systematic | | + | + | 8 | 1 | + | 3 | 3 | WT | - | - | A^+^B^+^C^WT^*cdtB*^-^ | x | - | - | - | - | - | SRR3115457 |
| DGF_0006 | | | DGF_0006_11 | | Systematic | | + | + | 8 | 1 | + | 3 | 3 | WT | - | - | A^+^B^+^C^WT^*cdtB*^-^ |  | NA | | | | | |
| DGF_0011 | | | DGF_0011_01 | | Systematic | | + | - | 3 | 1 | - | - | - | - | + | - | absent | x | - | - | - | - | - | SRR3115458 |
| DGF_0011 | | | DGF_0011_02 | | Systematic | | + | - | 3 | 1 | - | - | - | - | + | - | absent | x | - | - | - | - | - | SRR3115459 |
| DGF_0011 | | | DGF_0011_03 | | Systematic | | + | - | 3 | 1 | - | - | - | - | + | - | absent | x | - | - | - | - | - | SRR3115460 |
| DGF_0011 | | | DGF_0011_04 | | Systematic | | + | - | 3 | 1 | - | - | - | - | + | - | absent | x | - | - | - | - | - | SRR3115461 |
| DGF_0011 | | | DGF_0011_05 | | Systematic | | + | - | 3 | 1 | - | - | - | - | + | - | absent | x | - | - | - | - | - | SRR3115462 |
| DGF_0011 | | | DGF_0011_06 | | Systematic | | + | - | 3 | 1 | - | - | - | - | + | - | absent |  | NA | | | | | |
| DGF_0011 | | | DGF_0011_07 | | Systematic | | + | - | 3 | 1 | - | - | - | - | + | - | absent |  |  |  |  |  |  |  |
| DGF_0011 | | | DGF_0011_08 | | Systematic | | + | - | 3 | 1 | - | - | - | - | + | - | absent |  |  |  |  |  |  |  |
| DGF_0011 | | | DGF_0011_09 | | Systematic | | + | - | 3 | 1 | - | - | - | - | + | - | absent |  |  |  |  |  |  |  |
| DGF_0011 | | | DGF_0011_10 | | Systematic | | + | - | 3 | 1 | - | - | - | - | + | - | absent |  |  |  |  |  |  |  |
| DGF_0027 | | | DGF_0027_01 | | Systematic | | + | + | 42 | 1 | + | 3 | 3 | WT | - | - | A^+^B^+^C^WT^*cdtB*^-^ |  |  |  |  |  |  |  |
| DGF_0027 | | | DGF_0027_02 | | Systematic | | + | + | 42 | 1 | + | 3 | 3 | WT | - | - | A^+^B^+^C^WT^*cdtB*^-^ |  |  |  |  |  |  |  |
| DGF_0027 | | | DGF_0027_03 | | Systematic | | + | + | 42 | 1 | + | 3 | 3 | WT | - | - | A^+^B^+^C^WT^*cdtB*^-^ |  |  |  |  |  |  |  |
| DGF_0027 | | | DGF_0027_04 | | Systematic | | + | + | 42 | 1 | + | 3 | 3 | WT | - | - | A^+^B^+^C^WT^*cdtB*^-^ |  |  |  |  |  |  |  |
| DGF_0027 | | | DGF_0027_05 | | Systematic | | + | + | 42 | 1 | + | 3 | 3 | WT | - | - | A^+^B^+^C^WT^*cdtB*^-^ |  |  |  |  |  |  |  |
| DGF_0027 | | | DGF_0027_06 | | Systematic | | + | + | 42 | 1 | + | 3 | 3 | WT | - | - | A^+^B^+^C^WT^*cdtB*^-^ |  |  |  |  |  |  |  |
| DGF_0027 | | | DGF_0027_07 | | Systematic | | + | + | 42 | 1 | + | 3 | 3 | WT | - | - | A^+^B^+^C^WT^*cdtB*^-^ |  |  |  |  |  |  |  |
| DGF_0027 | | | DGF_0027_08 | | Systematic | | + | + | 42 | 1 | + | 3 | 3 | WT | - | - | A^+^B^+^C^WT^*cdtB*^-^ |  |  |  |  |  |  |  |
| DGF_0027 | | | DGF_0027_09 | | Systematic | | + | + | 42 | 1 | + | 3 | 3 | WT | - | - | A^+^B^+^C^WT^*cdtB*^-^ | x | - | - | - | - | - | SRR3115463 |
| DGF_0027 | | | DGF_0027_10 | | Systematic | | + | + | 42 | 1 | + | NA | 3 | WT | - | - | A^+^B^+^C^WT^*cdtB*^-^ |  | NA | | | | | |
| DGF_0034 | | | DGF_0034_01 | | Systematic | | + | - | 3 | 1 | - | - | - | - | + | - | absent | x | - | - | - | - | - | SRR3115464 |
| DGF_0034 | | | DGF_0034_02 | | Systematic | | + | - | 3 | 1 | - | - | - | - | + | - | absent |  | NA | | | | | |
| DGF_0034 | | | DGF_0034_03 | | Systematic | | + | - | 3 | 1 | - | - | - | - | + | - | absent |  |  |  |  |  |  |  |
| DGF_0034 | | | DGF_0034_04 | | Systematic | | + | - | 3 | 1 | - | - | - | - | + | - | absent |  |  |  |  |  |  |  |
| DGF_0034 | | | DGF_0034_05 | | Systematic | | + | - | 3 | 1 | - | - | - | - | + | - | absent |  |  |  |  |  |  |  |
| DGF_0034 | | | DGF_0034_07 | | Systematic | | + | - | 3 | 1 | - | - | - | - | + | - | absent |  |  |  |  |  |  |  |
| DGF_0034 | | | DGF_0034_08 | | Systematic | | + | - | 3 | 1 | - | - | - | - | + | - | absent |  |  |  |  |  |  |  |
| DGF_0036 | | | DGF_0036_01 | | Systematic | | + | - | 15 | 1 | - | - | - | - | + | - | absent |  |  |  |  |  |  |  |
| DGF_0036 | | | DGF_0036_02 | | Systematic | | + | - | 15 | 1 | - | - | - | - | + | - | absent |  |  |  |  |  |  |  |
| DGF_0036 | | | DGF_0036_03 | | Systematic | | + | - | 15 | 1 | - | - | - | - | + | - | absent | x | - | - | - | - | - | SRR3115465 |
| DGF_0036 | | | DGF_0036_04 | | Systematic | | + | - | 15 | 1 | - | - | - | - | + | - | absent |  | NA | | | | | |
| DGF_0036 | | | DGF_0036_05 | | Systematic | | + | - | 15 | 1 | - | - | - | - | + | - | absent |  |  |  |  |  |  |  |
| DGF_0036 | | | DGF_0036_06 | | Systematic | | + | - | 15 | 1 | - | - | - | - | + | - | absent |  |  |  |  |  |  |  |
| DGF_0036 | | | DGF_0036_07 | | Systematic | | + | - | 15 | 1 | - | - | - | - | + | - | absent |  |  |  |  |  |  |  |
| DGF_0036 | | | DGF_0036_08 | | Systematic | | + | - | 15 | 1 | - | - | - | - | + | - | absent |  |  |  |  |  |  |  |
| DGF_0036 | | | DGF_0036_09 | | Systematic | | + | - | 15 | 1 | - | - | - | - | + | - | absent |  |  |  |  |  |  |  |
| DGF_0036 | | | DGF_0036_10 | | Systematic | | + | - | 15 | 1 | - | - | - | - | + | - | absent |  |  |  |  |  |  |  |
| DGF_0036 | | | DGF_0036_11 | | Systematic | | + | + | 42 | 1 | + | 3 | 3 | WT | - | - | A^+^B^+^C^WT^*cdtB*^-^ | x | - | - | - | - | - | SRR3115466 |
| DGF_0036 | | | DGF_0036_12 | | Systematic | | + | + | 42 | 1 | + | 3 | 3 | WT | - | - | A^+^B^+^C^WT^*cdtB*^-^ |  | NA | | | | | |
| DGF_0036 | | | DGF_0036_13 | | Systematic | | + | - | 15 | 1 | - | - | - | - | + | - | absent |  |  |  |  |  |  |  |
| DGF_0036 | | | DGF_0036_14 | | Systematic | | + | + | 42 | 1 | + | 3 | 3 | WT | - | - | A^+^B^+^C^WT^*cdtB*^-^ | x | - | - | - | - | - | SRR3115467 |
| DGF_0036 | | | DGF_0036_15 | | Systematic | | + | + | 42 | 1 | + | 3 | 3 | WT | - | - | A^+^B^+^C^WT^*cdtB*^-^ |  | NA | | | | | |
| DGF_0040 | | | DGF_0040_01 | | Systematic | | + | - | 15 | 1 | - | - | - | - | + | - | absent |  |  |  |  |  |  |  |
| DGF_0040 | | | DGF_0040_02 | | Systematic | | + | - | 15 | 1 | - | - | - | - | + | - | absent |  |  |  |  |  |  |  |
| DGF_0040 | | | DGF_0040_03 | | Systematic | | + | - | 15 | 1 | - | - | - | - | + | - | absent |  |  |  |  |  |  |  |
| DGF_0040 | | | DGF_0040_04 | | Systematic | | + | - | 15 | 1 | - | - | - | - | + | - | absent | x | - | - | - | - | - | SRR3115468 |
| DGF_0040 | | | DGF_0040_05 | | Systematic | | + | - | 15 | 1 | - | - | - | - | + | - | absent |  | NA | | | | | |
| DGF_0040 | | | DGF_0040_06 | | Systematic | | + | - | 15 | 1 | - | - | - | - | + | - | absent |  |  |  |  |  |  |  |
| DGF_0040 | | | DGF_0040_07 | | Systematic | | + | - | 15 | 1 | - | - | - | - | + | - | absent |  |  |  |  |  |  |  |
| DGF_0040 | | | DGF_0040_08 | | Systematic | | + | - | 15 | 1 | - | - | - | - | + | - | absent |  |  |  |  |  |  |  |
| DGF_0040 | | | DGF_0040_09 | | Systematic | | + | - | 15 | 1 | - | - | - | - | + | - | absent |  |  |  |  |  |  |  |
| DGF_0040 | | | DGF_0040_10 | | Systematic | | + | - | 15 | 1 | - | - | - | - | + | - | absent |  |  |  |  |  |  |  |
| DGF_0048 | | | DGF_0048_01 | | Systematic | | + | + | 42 | 1 | + | 3 | 3 | WT | - | - | A^+^B^+^C^WT^*cdtB*^-^ | x | - | - | - | - | - | SRR3115469 |
| DGF_0048 | | | DGF_0048_02 | | Systematic | | + | + | 42 | 1 | + | 3 | 3 | WT | - | - | A^+^B^+^C^WT^*cdtB*^-^ | x | - | - | - | - | - | SRR3115470 |
| DGF_0048 | | | DGF_0048_03 | | Systematic | | + | + | 42 | 1 | + | 3 | 3 | WT | - | - | A^+^B^+^C^WT^*cdtB*^-^ |  | NA | | | | | |
| DGF_0048 | | | DGF_0048_04 | | Systematic | | + | + | 42 | 1 | + | 3 | 3 | WT | - | - | A^+^B^+^C^WT^*cdtB*^-^ | x | - | - | - | - | - | SRR3115471 |
| DGF_0048 | | | DGF_0048_05 | | Systematic | | + | + | 42 | 1 | + | 3 | 3 | WT | - | - | A^+^B^+^C^WT^*cdtB*^-^ | x | - | - | - | - | - | SRR3115472 |
| DGF_0048 | | | DGF_0048_06 | | Systematic | | + | + | 42 | 1 | + | 3 | 3 | WT | - | - | A^+^B^+^C^WT^*cdtB*^-^ | x | - | - | - | - | - | SRR3115473 |
| DGF_0048 | | | DGF_0048_07 | | Systematic | | + | + | 6 | 1 | + | 3 | 2 | WT | - | - | A^+^B^+^C^WT^*cdtB*^-^ | x | - | - | - | - | - | SRR3115475 |
| DGF_0048 | | | DGF_0048_08 | | Systematic | | + | + | 42 | 1 | + | 3 | 3 | WT | - | - | A^+^B^+^C^WT^*cdtB*^-^ | x | - | - | - | - | - | SRR3115474 |
| DGF_0048 | | | DGF_0048_11 | | Systematic | | + | + | 42 | 1 | + | 3 | 3 | WT | - | - | A^+^B^+^C^WT^*cdtB*^-^ |  | NA | | | | | |
| DGF_0048 | | | DGF_0048_12 | | Systematic | | + | + | 42 | 1 | + | 3 | 3 | WT | - | - | A^+^B^+^C^WT^*cdtB*^-^ |  |  |  |  |  |  |  |
| DGF_0048 | | | DGF_0048_13 | | Systematic | | + | + | 42 | 1 | + | 3 | 3 | WT | - | - | A^+^B^+^C^WT^*cdtB*^-^ |  |  |  |  |  |  |  |
| DGF_0048 | | | DGF_0048_14 | | Systematic | | + | + | 42 | 1 | + | 3 | 3 | WT | - | - | A^+^B^+^C^WT^*cdtB*^-^ |  |  |  |  |  |  |  |
| DGF_0048 | | | DGF_0048_15 | | Systematic | | + | + | 42 | 1 | + | 3 | 3 | WT | - | - | A^+^B^+^C^WT^*cdtB*^-^ |  |  |  |  |  |  |  |
| DGF_0050 | | | DGF_0050_01 | | Systematic | | + | + | 42 | 1 | + | 3 | 3 | WT | - | - | A^+^B^+^C^WT^*cdtB*^-^ |  |  |  |  |  |  |  |
| DGF_0050 | | | DGF_0050_03 | | Systematic | | + | + | 42 | 1 | + | 3 | 3 | WT | - | - | A^+^B^+^C^WT^*cdtB*^-^ |  |  |  |  |  |  |  |
| DGF_0050 | | | DGF_0050_04 | | Systematic | | + | + | 42 | 1 | + | 3 | 3 | WT | - | - | A^+^B^+^C^WT^*cdtB*^-^ |  |  |  |  |  |  |  |
| DGF_0050 | | | DGF_0050_05 | | Systematic | | + | + | 42 | 1 | + | 3 | 3 | WT | - | - | A^+^B^+^C^WT^*cdtB*^-^ |  |  |  |  |  |  |  |
| DGF_0050 | | | DGF_0050_18 | | Systematic | | + | + | 42 | 1 | + | 3 | 3 | WT | - | - | A^+^B^+^C^WT^*cdtB*^-^ |  |  |  |  |  |  |  |
| DGF_0050 | | | DGF_0050_19 | | Systematic | | + | + | 42 | 1 | + | 3 | 3 | WT | - | - | A^+^B^+^C^WT^*cdtB*^-^ |  |  |  |  |  |  |  |
| DGF_0050 | | | DGF_0050_20 | | Systematic | | + | + | 42 | 1 | + | 3 | 3 | WT | - | - | A^+^B^+^C^WT^*cdtB*^-^ |  |  |  |  |  |  |  |
| DGF_0050 | | | DGF_0050_21 | | Systematic | | + | + | 42 | 1 | + | 3 | 3 | WT | - | - | A^+^B^+^C^WT^*cdtB*^-^ |  |  |  |  |  |  |  |
| DGF_0050 | | | DGF_0050_22 | | Systematic | | + | + | 42 | 1 | + | 3 | 3 | WT | - | - | A^+^B^+^C^WT^*cdtB*^-^ |  |  |  |  |  |  |  |
| DGF_0050 | | | DGF_0050_23 | | Systematic | | + | + | 42 | 1 | + | 3 | 3 | WT | - | - | A^+^B^+^C^WT^*cdtB*^-^ |  |  |  |  |  |  |  |
| DGF_0050 | | | DGF_0050_24 | | Systematic | | + | + | 42 | 1 | + | 3 | 3 | WT | - | - | A^+^B^+^C^WT^*cdtB*^-^ |  |  |  |  |  |  |  |
| DGF_0050 | | | DGF_0050_25 | | Systematic | | + | + | 42 | 1 | + | 3 | 3 | WT | - | - | A^+^B^+^C^WT^*cdtB*^-^ |  |  |  |  |  |  |  |
| DGF_0050 | | | DGF_0050_26 | | Systematic | | + | + | 42 | 1 | + | 3 | 3 | WT | - | - | A^+^B^+^C^WT^*cdtB*^-^ |  |  |  |  |  |  |  |
| DGF_0050 | | | DGF_0050_27 | | Systematic | | + | + | 42 | 1 | + | 3 | 3 | WT | - | - | A^+^B^+^C^WT^*cdtB*^-^ |  |  |  |  |  |  |  |
| DGF_0050 | | | DGF_0050_28 | | Systematic | | + | + | 42 | 1 | + | 3 | 3 | WT | - | - | A^+^B^+^C^WT^*cdtB*^-^ | x | - | - | - | - | - | SRR3115476 |
| DGF_0050 | | | DGF_0050_29 | | Systematic | | + | + | 42 | 1 | + | 3 | 3 | WT | - | - | A^+^B^+^C^WT^*cdtB*^-^ |  | NA | | | | | |
| DGF_0050 | | | DGF_0050_30 | | Systematic | | + | + | 42 | 1 | + | 3 | 3 | WT | - | - | A^+^B^+^C^WT^*cdtB*^-^ |  |  |  |  |  |  |  |
| DGF_0059 | | | DGF_0059_01 | | Systematic | | + | - | 3 | 1 | - | - | - | - | + | - | absent | x | - | - | - | - | - | SRR3115477 |
| DGF_0059 | | | DGF_0059_02 | | Systematic | | + | - | 3 | 1 | - | - | - | - | + | - | absent |  | NA | | | | | |
| DGF_0059 | | | DGF_0059_03 | | Systematic | | + | - | 3 | 1 | - | - | - | - | + | - | absent |  |  |  |  |  |  |  |
| DGF_0059 | | | DGF_0059_04 | | Systematic | | + | - | 3 | 1 | - | - | - | - | + | - | absent |  |  |  |  |  |  |  |
| DGF_0059 | | | DGF_0059_05 | | Systematic | | + | - | 3 | 1 | - | - | - | - | + | - | absent |  |  |  |  |  |  |  |
| DGF_0059 | | | DGF_0059_06 | | Systematic | | + | - | 3 | 1 | - | - | - | - | + | - | absent |  |  |  |  |  |  |  |
| DGF_0059 | | | DGF_0059_07 | | Systematic | | + | - | 3 | 1 | - | - | - | - | + | - | absent |  |  |  |  |  |  |  |
| DGF_0059 | | | DGF_0059_08 | | Systematic | | + | - | 3 | 1 | - | - | - | - | + | - | absent |  |  |  |  |  |  |  |
| DGF_0059 | | | DGF_0059_09 | | Systematic | | + | - | 3 | 1 | - | - | - | - | + | - | absent |  |  |  |  |  |  |  |
| DGF_0059 | | | DGF_0059_10 | | Systematic | | + | - | 3 | 1 | - | - | - | - | + | - | absent |  |  |  |  |  |  |  |
| DGF_0062 | | | DGF_0062_03 | | Systematic | | + | + | 28 | 1 | + | 3 | 7 | Δ18 | - | - | A^+^B^+^C^Δ18^*cdtB*^-^ | x | - | - | - | - | - | SRR3115478 |
| DGF_0062 | | | DGF_0062_04 | | Systematic | | + | + | 28 | 1 | + | 3 | 7 | Δ18 | - | - | A^+^B^+^C^Δ18^*cdtB*^-^ | x | - | - | - | - | - | SRR3115479 |
| DGF_0062 | | | DGF_0062_11 | | Systematic | | + | + | 28 | 1 | + | 3 | 7 | Δ18 | - | - | A^+^B^+^C^Δ18^*cdtB*^-^ |  | NA | | | | | |
| DGF_0062 | | | DGF_0062_13 | | Systematic | | + | + | 28 | 1 | + | 3 | 7 | Δ18 | - | - | A^+^B^+^C^Δ18^*cdtB*^-^ |  |  |  |  |  |  |  |
| DGF_0062 | | | DGF_0062_14 | | Systematic | | + | + | 28 | 1 | + | 3 | 7 | Δ18 | - | - | A^+^B^+^C^Δ18^*cdtB*^-^ |  |  |  |  |  |  |  |
| DGF_0062 | | | DGF_0062_15 | | Systematic | | + | + | 28 | 1 | + | 3 | 7 | Δ18 | - | - | A^+^B^+^C^Δ18^*cdtB*^-^ |  |  |  |  |  |  |  |
| DGF_0062 | | | DGF_0062_16 | | Systematic | | + | + | 28 | 1 | + | 3 | 7 | Δ18 | - | - | A^+^B^+^C^Δ18^*cdtB*^-^ |  |  |  |  |  |  |  |
| DGF_0062 | | | DGF_0062_17 | | Systematic | | + | + | 28 | 1 | + | 3 | 7 | Δ18 | - | - | A^+^B^+^C^Δ18^*cdtB*^-^ |  |  |  |  |  |  |  |
| DGF_0062 | | | DGF_0062_18 | | Systematic | | + | + | 28 | 1 | + | 3 | 7 | Δ18 | - | - | A^+^B^+^C^Δ18^*cdtB*^-^ |  |  |  |  |  |  |  |
| DGF_0062 | | | DGF_0062_20 | | Systematic | | + | + | 28 | 1 | + | 3 | 7 | Δ18 | - | - | A^+^B^+^C^Δ18^*cdtB*^-^ |  |  |  |  |  |  |  |
| DGF_0062 | | | DGF_0062_21 | | Systematic | | + | + | 28 | 1 | + | 3 | 7 | Δ18 | - | - | A^+^B^+^C^Δ18^*cdtB*^-^ |  |  |  |  |  |  |  |
| DGF_0062 | | | DGF_0062_22 | | Systematic | | + | + | 28 | 1 | + | 3 | 7 | Δ18 | - | - | A^+^B^+^C^Δ18^*cdtB*^-^ |  |  |  |  |  |  |  |
| DGF_0062 | | | DGF_0062_23 | | Systematic | | + | - | 15 | 1 | - | - | - | - | + | - | absent | x | - | - | - | - | - | SRR3115480 |
| DGF_0062 | | | DGF_0062_24 | | Systematic | | + | - | 15 | 1 | - | - | - | - | + | - | absent |  | NA | | | | | |
| DGF_0062 | | | DGF_0062_25 | | Systematic | | + | - | 15 | 1 | - | - | - | - | + | - | absent |  |  |  |  |  |  |  |
| DGF_0062 | | | DGF_0062_26 | | Systematic | | + | - | 15 | 1 | - | - | - | - | + | - | absent |  |  |  |  |  |  |  |
| DGF_0062 | | | DGF_0062_27 | | Systematic | | + | - | 15 | 1 | - | - | - | - | + | - | absent |  |  |  |  |  |  |  |
| DGF_0063 | | | DGF_0063_01 | | Systematic | | + | - | 29 | 1 | - | - | - | - | + | - | absent |  |  |  |  |  |  |  |
| DGF_0063 | | | DGF_0063_02 | | Systematic | | + | - | 29 | 1 | - | - | - | - | + | - | absent |  |  |  |  |  |  |  |
| DGF_0063 | | | DGF_0063_03 | | Systematic | | + | - | 29 | 1 | - | - | - | - | + | - | absent |  |  |  |  |  |  |  |
| DGF_0063 | | | DGF_0063_04 | | Systematic | | + | - | 29 | 1 | - | - | - | - | + | - | absent | x | - | - | - | - | - | SRR3115481 |
| DGF_0063 | | | DGF_0063_05 | | Systematic | | + | - | 29 | 1 | - | - | - | - | + | - | absent |  | NA | | | | | |
| DGF_0063 | | | DGF_0063_06 | | Systematic | | + | - | 29 | 1 | - | - | - | - | + | - | absent |  |  |  |  |  |  |  |
| DGF_0063 | | | DGF_0063_07 | | Systematic | | + | - | 29 | 1 | - | - | - | - | + | - | absent |  |  |  |  |  |  |  |
| DGF_0063 | | | DGF_0063_08 | | Systematic | | + | - | 29 | 1 | - | - | - | - | + | - | absent |  |  |  |  |  |  |  |
| DGF_0063 | | | DGF_0063_09 | | Systematic | | + | - | 29 | 1 | - | - | - | - | + | - | absent |  |  |  |  |  |  |  |
| DGF_0063 | | | DGF_0063_10 | | Systematic | | + | - | 29 | 1 | - | - | - | - | + | - | absent |  |  |  |  |  |  |  |
| **DGF_0065*** | | | NA | | Systematic | | + | + | NA | NA | + | NA | 31 | WT | - | - | A^+^B^+^C^WT^*cdtB*^-^ |  |  |  |  |  |  |  |
| **DGF_0088†** | | | NA | | Systematic | | + | + | NA | NA | + | 3 | 31 | WT | - | - | A^+^B^+^C^WT^*cdtB*^-^ |  |  |  |  |  |  |  |
| DGF_0092 | | | DGF_0092_01 | | Systematic | | + | + | 2 | 1 | + | 3 | 4 | WT | - | - | A^+^B^+^C^WT^*cdtB*^-^ | x | - | - | - | - | - | SRR3115482 |
| DGF_0092 | | | DGF_0092_02 | | Systematic | | + | + | 2 | 1 | + | 3 | 4 | WT | - | - | A^+^B^+^C^WT^*cdtB*^-^ |  | NA | | | | | |
| DGF_0092 | | | DGF_0092_03 | | Systematic | | + | + | 2 | 1 | + | 3 | 4 | WT | - | - | A^+^B^+^C^WT^*cdtB*^-^ |  |  |  |  |  |  |  |
| DGF_0092 | | | DGF_0092_04 | | Systematic | | + | + | 2 | 1 | + | 3 | 4 | WT | - | - | A^+^B^+^C^WT^*cdtB*^-^ |  |  |  |  |  |  |  |
| DGF_0092 | | | DGF_0092_05 | | Systematic | | + | + | 2 | 1 | + | 3 | 4 | WT | - | - | A^+^B^+^C^WT^*cdtB*^-^ |  |  |  |  |  |  |  |
| DGF_0092 | | | DGF_0092_06 | | Systematic | | + | + | 2 | 1 | + | 3 | 4 | WT | - | - | A^+^B^+^C^WT^*cdtB*^-^ |  |  |  |  |  |  |  |
| DGF_0092 | | | DGF_0092_07 | | Systematic | | + | + | 2 | 1 | + | 3 | 4 | WT | - | - | A^+^B^+^C^WT^*cdtB*^-^ |  |  |  |  |  |  |  |
| DGF_0092 | | | DGF_0092_08 | | Systematic | | + | + | 2 | 1 | + | 3 | 4 | WT | - | - | A^+^B^+^C^WT^*cdtB*^-^ |  |  |  |  |  |  |  |
| DGF_0092 | | | DGF_0092_09 | | Systematic | | + | + | 2 | 1 | + | 3 | 4 | WT | - | - | A^+^B^+^C^WT^*cdtB*^-^ |  |  |  |  |  |  |  |
| DGF_0092 | | | DGF_0092_10 | | Systematic | | + | + | 2 | 1 | + | 3 | 4 | WT | - | - | A^+^B^+^C^WT^*cdtB*^-^ |  |  |  |  |  |  |  |
| DGF_0103 | | | DGF_0103_01 | | Systematic | | + | + | 2 | 1 | + | 3 | 4 | WT | - | - | A^+^B^+^C^WT^*cdtB*^-^ |  |  |  |  |  |  |  |
| DGF_0103 | | | DGF_0103_02 | | Systematic | | + | + | 2 | 1 | + | 3 | 4 | WT | - | - | A^+^B^+^C^WT^*cdtB*^-^ |  |  |  |  |  |  |  |
| DGF_0103 | | | DGF_0103_03 | | Systematic | | + | + | 2 | 1 | + | 3 | 4 | WT | - | - | A^+^B^+^C^WT^*cdtB*^-^ |  |  |  |  |  |  |  |
| DGF_0103 | | | DGF_0103_04 | | Systematic | | + | + | 2 | 1 | + | 3 | 4 | WT | - | - | A^+^B^+^C^WT^*cdtB*^-^ | x | - | - | - | - | - | SRR3115483 |
| DGF_0103 | | | DGF_0103_05 | | Systematic | | + | + | 2 | 1 | + | 3 | 4 | WT | - | - | A^+^B^+^C^WT^*cdtB*^-^ |  | NA | | | | | |
| DGF_0103 | | | DGF_0103_06 | | Systematic | | + | + | 2 | 1 | + | 3 | 4 | WT | - | - | A^+^B^+^C^WT^*cdtB*^-^ |  |  |  |  |  |  |  |
| DGF_0103 | | | DGF_0103_07 | | Systematic | | + | + | 2 | 1 | + | 3 | 4 | WT | - | - | A^+^B^+^C^WT^*cdtB*^-^ |  |  |  |  |  |  |  |
| DGF_0103 | | | DGF_0103_08 | | Systematic | | + | + | 2 | 1 | + | 3 | 4 | WT | - | - | A^+^B^+^C^WT^*cdtB*^-^ |  |  |  |  |  |  |  |
| **DGF_0112** | | | NA | | Systematic | | + | + | 42 | 1 | + | 3 | 3 | WT | - | - | A^+^B^+^C^WT^*cdtB*^-^ |  |  |  |  |  |  |  |
| DGF_0113 | | | DGF_0113_01 | | Systematic | | + | + | 2 | 1 | + | 3 | 4 | WT | - | - | A^+^B^+^C^WT^*cdtB*^-^ | x | - | - | - | - | - | SRR3115486 |
| DGF_0113 | | | DGF_0113_02 | | Systematic | | + | - | 15 | 1 | - | - | - | - | + | - | absent | x | - | - | - | - | - | SRR3115484 |
| DGF_0113 | | | DGF_0113_03 | | Systematic | | + | - | 15 | 1 | - | - | - | - | + | - | absent | x | - | - | - | - | - | SRR3115485 |
| DGF_0113 | | | DGF_0113_04 | | Systematic | | + | - | 15 | 1 | - | - | - | - | + | - | absent |  | NA | | | | | |
| DGF_0113 | | | DGF_0113_05 | | Systematic | | + | - | 15 | 1 | - | - | - | - | + | - | absent |  |  |  |  |  |  |  |
| DGF_0113 | | | DGF_0113_06 | | Systematic | | + | + | 2 | 1 | + | 3 | 4 | WT | - | - | A^+^B^+^C^WT^*cdtB*^-^ |  |  |  |  |  |  |  |
| DGF_0113 | | | DGF_0113_07 | | Systematic | | + | + | 10 | 1 | + | 3 | 7 | Δ18 | - | - | A^+^B^+^C^Δ18^*cdtB*^-^ | x | - | - | - | - | - | SRR3115487 |
| DGF_0113 | | | DGF_0113_08 | | Systematic | | + | + | 2 | 1 | + | 3 | 4 | WT | - | - | A^+^B^+^C^WT^*cdtB*^-^ |  | NA | | | | | |
| DGF_0113 | | | DGF_0113_09 | | Systematic | | + | + | 10 | 1 | + | 3 | 7 | Δ18 | - | - | A^+^B^+^C^Δ18^*cdtB*^-^ |  |  |  |  |  |  |  |
| DGF_0113 | | | DGF_0113_10 | | Systematic | | + | + | 10 | 1 | + | 3 | 7 | Δ18 | - | - | A^+^B^+^C^Δ18^*cdtB*^-^ |  |  |  |  |  |  |  |
| DGF_0120 | | | DGF_0120_01 | | Systematic | | + | - | 3 | 1 | - | - | - | - | + | - | absent | x | - | - | - | - | - | SRR3115488 |
| DGF_0120 | | | DGF_0120_02 | | Systematic | | + | - | 3 | 1 | - | - | - | - | + | - | absent |  | NA | | | | | |
| DGF_0120 | | | DGF_0120_03 | | Systematic | | + | - | 3 | 1 | - | - | - | - | + | - | absent | x | - | - | - | - | - | SRR3115489 |
| DGF_0120 | | | DGF_0120_04 | | Systematic | | + | - | 3 | 1 | - | - | - | - | + | - | absent |  | NA | | | | | |
| DGF_0120 | | | DGF_0120_05 | | Systematic | | + | - | 3 | 1 | - | - | - | - | + | - | absent |  |  |  |  |  |  |  |
| DGF_0120 | | | DGF_0120_06 | | Systematic | | + | - | 3 | 1 | - | - | - | - | + | - | absent |  |  |  |  |  |  |  |
| DGF_0120 | | | DGF_0120_07 | | Systematic | | + | - | 3 | 1 | - | - | - | - | + | - | absent |  |  |  |  |  |  |  |
| DGF_0120 | | | DGF_0120_08 | | Systematic | | + | - | 3 | 1 | - | - | - | - | + | - | absent |  |  |  |  |  |  |  |
| DGF_0120 | | | DGF_0120_09 | | Systematic | | + | - | 3 | 1 | - | - | - | - | + | - | absent |  |  |  |  |  |  |  |
| DGF_0120 | | | DGF_0120_10 | | Systematic | | + | - | 3 | 1 | - | - | - | - | + | - | absent |  |  |  |  |  |  |  |
| DGF_0132 | | | DGF_0132_01 | | Systematic | | + | + | 110 | 1 | + | 3 | 31 | WT | - | - | A^+^B^+^C^WT^*cdtB*^-^ |  |  |  |  |  |  |  |
| DGF_0132 | | | DGF_0132_02 | | Systematic | | + | + | 110 | 1 | + | 3 | 31 | WT | - | - | A^+^B^+^C^WT^*cdtB*^-^ |  |  |  |  |  |  |  |
| DGF_0132 | | | DGF_0132_03 | | Systematic | | + | + | 110 | 1 | + | 3 | 31 | WT | - | - | A^+^B^+^C^WT^*cdtB*^-^ |  |  |  |  |  |  |  |
| DGF_0132 | | | DGF_0132_04 | | Systematic | | + | + | 110 | 1 | + | 3 | 31 | WT | - | - | A^+^B^+^C^WT^*cdtB*^-^ |  |  |  |  |  |  |  |
| DGF_0132 | | | DGF_0132_05 | | Systematic | | + | + | 110 | 1 | + | 3 | 31 | WT | - | - | A^+^B^+^C^WT^*cdtB*^-^ |  |  |  |  |  |  |  |
| DGF_0132 | | | DGF_0132_06 | | Systematic | | + | + | 110 | 1 | + | 3 | 31 | WT | - | - | A^+^B^+^C^WT^*cdtB*^-^ |  |  |  |  |  |  |  |
| DGF_0132 | | | DGF_0132_07 | | Systematic | | + | + | 110 | 1 | + | 3 | 31 | WT | - | - | A^+^B^+^C^WT^*cdtB*^-^ | x | - | - | - | - | - | SRR3115490 |
| DGF_0132 | | | DGF_0132_08 | | Systematic | | + | + | 110 | 1 | + | 3 | 31 | WT | - | - | A^+^B^+^C^WT^*cdtB*^-^ |  | NA | | | | | |
| DGF_0134 | | | DGF_0134_01 | | Systematic | | + | + | 2 | 1 | + | 3 | 4 | WT | - | - | A^+^B^+^C^WT^*cdtB*^-^ |  |  |  |  |  |  |  |
| DGF_0134 | | | DGF_0134_02 | | Systematic | | + | + | 2 | 1 | + | 3 | 4 | WT | - | - | A^+^B^+^C^WT^*cdtB*^-^ | x | - | - | - | - | - | SRR3115491 |
| DGF_0134 | | | DGF_0134_03 | | Systematic | | + | + | 2 | 1 | + | 3 | 4 | WT | - | - | A^+^B^+^C^WT^*cdtB*^-^ |  | NA | | | | | |
| DGF_0134 | | | DGF_0134_04 | | Systematic | | + | + | 2 | 1 | + | 3 | 4 | WT | - | - | A^+^B^+^C^WT^*cdtB*^-^ |  |  |  |  |  |  |  |
| DGF_0134 | | | DGF_0134_05 | | Systematic | | + | + | 2 | 1 | + | 3 | 4 | WT | - | - | A^+^B^+^C^WT^*cdtB*^-^ |  |  |  |  |  |  |  |
| DGF_0134 | | | DGF_0134_06 | | Systematic | | + | + | 2 | 1 | + | 3 | 4 | WT | - | - | A^+^B^+^C^WT^*cdtB*^-^ |  |  |  |  |  |  |  |
| DGF_0134 | | | DGF_0134_07 | | Systematic | | + | + | 2 | 1 | + | 3 | 4 | WT | - | - | A^+^B^+^C^WT^*cdtB*^-^ |  |  |  |  |  |  |  |
| DGF_0134 | | | DGF_0134_08 | | Systematic | | + | + | 2 | 1 | + | 3 | 4 | WT | - | - | A^+^B^+^C^WT^*cdtB*^-^ |  |  |  |  |  |  |  |
| DGF_0134 | | | DGF_0134_09 | | Systematic | | + | + | 2 | 1 | + | 3 | 4 | WT | - | - | A^+^B^+^C^WT^*cdtB*^-^ |  |  |  |  |  |  |  |
| DGF_0134 | | | DGF_0134_10 | | Systematic | | + | + | 2 | 1 | + | 3 | 4 | WT | - | - | A^+^B^+^C^WT^*cdtB*^-^ |  |  |  |  |  |  |  |
| DGF_0134 | | | DGF_0134_11 | | Systematic | | + | + | 2 | 1 | + | 3 | 4 | WT | - | - | A^+^B^+^C^WT^*cdtB*^-^ |  |  |  |  |  |  |  |
| DGF_0134 | | | DGF_0134_12 | | Systematic | | + | + | 2 | 1 | + | 3 | 4 | WT | - | - | A^+^B^+^C^WT^*cdtB*^-^ |  |  |  |  |  |  |  |
| DGF_0134 | | | DGF_0134_13 | | Systematic | | + | + | 2 | 1 | + | 3 | 4 | WT | - | - | A^+^B^+^C^WT^*cdtB*^-^ |  |  |  |  |  |  |  |
| DGF_0134 | | | DGF_0134_14 | | Systematic | | + | - | 15 | 1 | - | - | - | - | + | - | absent | x | - | + | - | - | - | SRR3115492 |
| DGF_0134 | | | DGF_0134_15 | | Systematic | | + | + | 2 | 1 | + | 3 | 4 | WT | - | - | A^+^B^+^C^WT^*cdtB*^-^ |  | NA | | | | | |
| **DGF_0148** | | | NA | | Systematic | | + | - | 3 | 1 | - | - | - | - | + | - | absent |  |  |  |  |  |  |  |
| DGF_0153 | | | DGF_0153_01 | | Systematic | | + | - | 3 | 1 | - | - | - | - | + | - | absent | x | - | - | - | - | - | SRR3115493 |
| DGF_0153 | | | DGF_0153_02 | | Systematic | | + | - | 3 | 1 | - | - | - | - | + | - | absent |  | NA | | | | | |
| DGF_0153 | | | DGF_0153_03 | | Systematic | | + | - | 3 | 1 | - | - | - | - | + | - | absent |  |  |  |  |  |  |  |
| DGF_0153 | | | DGF_0153_04 | | Systematic | | + | - | 3 | 1 | - | - | - | - | + | - | absent |  |  |  |  |  |  |  |
| DGF_0153 | | | DGF_0153_05 | | Systematic | | + | - | 3 | 1 | - | - | - | - | + | - | absent |  |  |  |  |  |  |  |
| DGF_0153 | | | DGF_0153_06 | | Systematic | | + | - | 3 | 1 | - | - | - | - | + | - | absent |  |  |  |  |  |  |  |
| DGF_0153 | | | DGF_0153_07 | | Systematic | | + | - | 3 | 1 | - | - | - | - | + | - | absent |  |  |  |  |  |  |  |
| DGF_0153 | | | DGF_0153_08 | | Systematic | | + | - | 3 | 1 | - | - | - | - | + | - | absent |  |  |  |  |  |  |  |
| DGF_0153 | | | DGF_0153_09 | | Systematic | | + | - | 3 | 1 | - | - | - | - | + | - | absent |  |  |  |  |  |  |  |
| DGF_0153 | | | DGF_0153_10 | | Systematic | | + | - | 3 | 1 | - | - | - | - | + | - | absent |  |  |  |  |  |  |  |
| DGF_0156 | | | DGF_0156_01 | | Systematic | | + | + | 42 | 1 | + | 3 | 3 | WT | - | - | A^+^B^+^C^WT^*cdtB*^-^ | x | - | - | - | - | - | SRR3115494 |
| DGF_0156 | | | DGF_0156_02 | | Systematic | | + | + | 42 | 1 | + | 3 | 3 | WT | - | - | A^+^B^+^C^WT^*cdtB*^-^ | x | - | - | - | - | - | SRR3115495 |
| **DGF_0157*** | | | NA | | Systematic | | + | - | NA | NA | - | - | - | - | + | - | absent |  | NA | | | | | |
| **DGF_0160** | | | NA | | Systematic | | + | - | 15 | 1 | - | - | - | - | + | - | absent |  |  |  |  |  |  |  |
| **DGF_0163** | | | NA | | Systematic | | + | + | 2 | 1 | + | 3 | 4 | WT | - | - | A^+^B^+^C^WT^*cdtB*^-^ |  |  |  |  |  |  |  |
| DGF_0172 | | | DGF_0172_01 | | Systematic | | + | - | 15 | 1 | - | - | - | - | + | - | absent | x | - | - | - | - | - | SRR3115496 |
| DGF_0172 | | | DGF_0172_02 | | Systematic | | + | - | 15 | 1 | - | - | - | - | + | - | absent |  | NA | | | | | |
| DGF_0172 | | | DGF_0172_03 | | Systematic | | + | - | 15 | 1 | - | - | - | - | + | - | absent |  |  |  |  |  |  |  |
| DGF_0172 | | | DGF_0172_04 | | Systematic | | + | - | 15 | 1 | - | - | - | - | + | - | absent |  |  |  |  |  |  |  |
| DGF_0172 | | | DGF_0172_05 | | Systematic | | + | - | 15 | 1 | - | - | - | - | + | - | absent |  |  |  |  |  |  |  |
| DGF_0172 | | | DGF_0172_06 | | Systematic | | + | - | 15 | 1 | - | - | - | - | + | - | absent |  |  |  |  |  |  |  |
| DGF_0172 | | | DGF_0172_07 | | Systematic | | + | - | 15 | 1 | - | - | - | - | + | - | absent |  |  |  |  |  |  |  |
| DGF_0172 | | | DGF_0172_08 | | Systematic | | + | - | 15 | 1 | - | - | - | - | + | - | absent |  |  |  |  |  |  |  |
| DGF_0172 | | | DGF_0172_09 | | Systematic | | + | - | 15 | 1 | - | - | - | - | + | - | absent |  |  |  |  |  |  |  |
| DGF_0173 | | | DGF_0173_01 | | Systematic | | + | - | 15 | 1 | - | - | - | - | + | - | absent | x | - | - | - | - | - | SRR3115497 |
| DGF_0173 | | | DGF_0173_02 | | Systematic | | + | - | 15 | 1 | - | - | - | - | + | - | absent |  | NA | | | | | |
| DGF_0173 | | | DGF_0173_03 | | Systematic | | + | - | 15 | 1 | - | - | - | - | + | - | absent |  |  |  |  |  |  |  |
| DGF_0173 | | | DGF_0173_04 | | Systematic | | + | - | 15 | 1 | - | - | - | - | + | - | absent |  |  |  |  |  |  |  |
| DGF_0173 | | | DGF_0173_05 | | Systematic | | + | - | 15 | 1 | - | - | - | - | + | - | absent |  |  |  |  |  |  |  |
| DGF_0173 | | | DGF_0173_06 | | Systematic | | + | - | 15 | 1 | - | - | - | - | + | - | absent |  |  |  |  |  |  |  |
| DGF_0173 | | | DGF_0173_07 | | Systematic | | + | - | 15 | 1 | - | - | - | - | + | - | absent |  |  |  |  |  |  |  |
| DGF_0173 | | | DGF_0173_08 | | Systematic | | + | - | 15 | 1 | - | - | - | - | + | - | absent |  |  |  |  |  |  |  |
| DGF_0173 | | | DGF_0173_09 | | Systematic | | + | - | 15 | 1 | - | - | - | - | + | - | absent |  |  |  |  |  |  |  |
| DGF_0173 | | | DGF_0173_10 | | Systematic | | + | - | 15 | 1 | - | - | - | - | + | - | absent |  |  |  |  |  |  |  |
| **DGF_0177** | | | NA | | Systematic | | + | + | 42 | 1 | + | 3 | 3 | WT | - | - | A^+^B^+^C^WT^*cdtB*^-^ |  |  |  |  |  |  |  |
| DGF_0179 | | | DGF_0179_01 | | Systematic | | + | + | 2 | 1 | + | 3 | 4 | WT | - | - | A^+^B^+^C^WT^*cdtB*^-^ |  |  |  |  |  |  |  |
| DGF_0196 | | | DGF_0196_01 | | Systematic | | + | + | 42 | 1 | + | 3 | 3 | WT | - | - | A^+^B^+^C^WT^*cdtB*^-^ |  |  |  |  |  |  |  |
| DGF_0196 | | | DGF_0196_05 | | Systematic | | + | + | 42 | 1 | + | 3 | 3 | WT | - | - | A^+^B^+^C^WT^*cdtB*^-^ |  |  |  |  |  |  |  |
| DGF_0196 | | | DGF_0196_06 | | Systematic | | + | + | 2 | 1 | + | 3 | 4 | WT | - | - | A^+^B^+^C^WT^*cdtB*^-^ |  |  |  |  |  |  |  |
| DGF_0196 | | | DGF_0196_07 | | Systematic | | + | + | 2 | 1 | + | 3 | 4 | WT | - | - | A^+^B^+^C^WT^*cdtB*^-^ |  |  |  |  |  |  |  |
| DGF_0196 | | | DGF_0196_08 | | Systematic | | + | + | 2 | 1 | + | 3 | 4 | WT | - | - | A^+^B^+^C^WT^*cdtB*^-^ |  |  |  |  |  |  |  |
| DGF_0196 | | | DGF_0196_09 | | Systematic | | + | + | 42 | 1 | + | 3 | 3 | WT | - | - | A^+^B^+^C^WT^*cdtB*^-^ |  |  |  |  |  |  |  |
| DGF_0196 | | | DGF_0196_10 | | Systematic | | + | + | 2 | 1 | + | 3 | 4 | WT | - | - | A^+^B^+^C^WT^*cdtB*^-^ | x | - | - | Thr-82→Ile | - | - | SRR3115498 |
| DGF_0196 | | | DGF_0196_11 | | Systematic | | + | + | 2 | 1 | + | 3 | 4 | WT | - | - | A^+^B^+^C^WT^*cdtB*^-^ |  | NA | | | | | |
| DGF_0196 | | | DGF_0196_12 | | Systematic | | + | + | 2 | 1 | + | 3 | 4 | WT | - | - | A^+^B^+^C^WT^*cdtB*^-^ |  |  |  |  |  |  |  |
| DGF_0196 | | | DGF_0196_13 | | Systematic | | + | + | 42 | 1 | + | 3 | 3 | WT | - | - | A^+^B^+^C^WT^*cdtB*^-^ | x | - | - | - | - | - | SRR3115499 |
| DGF_0196 | | | DGF_0196_14 | | Systematic | | + | + | 42 | 1 | + | 3 | 3 | WT | - | - | A^+^B^+^C^WT^*cdtB*^-^ |  | NA | | | | | |
| DGF_0196 | | | DGF_0196_15 | | Systematic | | + | + | 2 | 1 | + | 3 | 4 | WT | - | - | A^+^B^+^C^WT^*cdtB*^-^ |  |  |  |  |  |  |  |
| DGF_0196 | | | DGF_0196_16 | | Systematic | | + | + | 42 | 1 | + | 3 | 3 | WT | - | - | A^+^B^+^C^WT^*cdtB*^-^ |  |  |  |  |  |  |  |
| DGF_0196 | | | DGF_0196_17 | | Systematic | | + | + | 2 | 1 | + | 3 | 4 | WT | - | - | A^+^B^+^C^WT^*cdtB*^-^ |  |  |  |  |  |  |  |
| DGF_0196 | | | DGF_0196_18 | | Systematic | | + | + | 42 | 1 | + | 3 | 3 | WT | - | - | A^+^B^+^C^WT^*cdtB*^-^ |  |  |  |  |  |  |  |
| DGF_0196 | | | DGF_0196_19 | | Systematic | | + | + | 42 | 1 | + | 3 | 3 | WT | - | - | A^+^B^+^C^WT^*cdtB*^-^ |  |  |  |  |  |  |  |
| DGF_0196 | | | DGF_0196_20 | | Systematic | | + | + | 42 | 1 | + | 3 | 3 | WT | - | - | A^+^B^+^C^WT^*cdtB*^-^ |  |  |  |  |  |  |  |
| DGF_0196 | | | DGF_0196_21 | | Systematic | | + | + | 42 | 1 | + | 3 | 3 | WT | - | - | A^+^B^+^C^WT^*cdtB*^-^ |  |  |  |  |  |  |  |
| DGF_0196 | | | DGF_0196_23 | | Systematic | | + | + | 2 | 1 | + | 3 | 4 | WT | - | - | A^+^B^+^C^WT^*cdtB*^-^ |  |  |  |  |  |  |  |
| DGF_0199 | | | DGF_0199_01 | | Systematic | | + | - | 3 | 1 | - | - | - | - | + | - | absent |  |  |  |  |  |  |  |
| DGF_0199 | | | DGF_0199_02 | | Systematic | | + | - | 3 | 1 | - | - | - | - | + | - | absent | x | - | - | - | - | - | SRR3115500 |
| DGF_0199 | | | DGF_0199_03 | | Systematic | | + | - | 3 | 1 | - | - | - | - | + | - | absent |  | NA | | | | | |
| DGF_0199 | | | DGF_0199_08 | | Systematic | | + | - | 3 | 1 | - | - | - | - | + | - | absent |  |  |  |  |  |  |  |
| DGF_0199 | | | DGF_0199_10 | | Systematic | | + | - | 3 | 1 | - | - | - | - | + | - | absent |  |  |  |  |  |  |  |
| DGF_0199 | | | DGF_0199_11 | | Systematic | | + | - | 3 | 1 | - | - | - | - | + | - | absent |  |  |  |  |  |  |  |
| DGF_0201 | | | DGF_0201_01 | | Veterinary | | + | - | 26 | 1 | - | - | - | - | + | - | absent |  |  |  |  |  |  |  |
| DGF_0201 | | | DGF_0201_02 | | Veterinary | | + | - | 26 | 1 | - | - | - | - | + | - | absent |  |  |  |  |  |  |  |
| DGF_0201 | | | DGF_0201_03 | | Veterinary | | + | - | 26 | 1 | - | - | - | - | + | - | absent |  |  |  |  |  |  |  |
| DGF_0201 | | | DGF_0201_04 | | Veterinary | | + | - | 26 | 1 | - | - | - | - | + | - | absent |  |  |  |  |  |  |  |
| DGF_0201 | | | DGF_0201_05 | | Veterinary | | + | - | 26 | 1 | - | - | - | - | + | - | absent |  |  |  |  |  |  |  |
| DGF_0201 | | | DGF_0201_06 | | Veterinary | | + | - | 26 | 1 | - | - | - | - | + | - | absent |  |  |  |  |  |  |  |
| DGF_0201 | | | DGF_0201_07 | | Veterinary | | + | - | 26 | 1 | - | - | - | - | + | - | absent | x | + | + | - | - | - | SRR3115501 |
| DGF_0201 | | | DGF_0201_08 | | Veterinary | | + | - | 26 | 1 | - | - | - | - | + | - | absent |  | NA | | | | | |
| DGF_0201 | | | DGF_0201_09 | | Veterinary | | + | - | 26 | 1 | - | - | - | - | + | - | absent |  |  |  |  |  |  |  |
| DGF_0201 | | | DGF_0201_10 | | Veterinary | | + | - | 26 | 1 | - | - | - | - | + | - | absent |  |  |  |  |  |  |  |
| DGF_0205 | | | DGF_0205_01 | | Veterinary | | + | - | 3 | 1 | - | - | - | - | + | - | absent |  |  |  |  |  |  |  |
| DGF_0205 | | | DGF_0205_02 | | Veterinary | | + | - | 3 | 1 | - | - | - | - | + | - | absent |  |  |  |  |  |  |  |
| DGF_0205 | | | DGF_0205_03 | | Veterinary | | + | - | 3 | 1 | - | - | - | - | + | - | absent |  |  |  |  |  |  |  |
| DGF_0205 | | | DGF_0205_04 | | Veterinary | | + | - | 3 | 1 | - | - | - | - | + | - | absent |  |  |  |  |  |  |  |
| DGF_0205 | | | DGF_0205_05 | | Veterinary | | + | - | 3 | 1 | - | - | - | - | + | - | absent |  |  |  |  |  |  |  |
| DGF_0205 | | | DGF_0205_06 | | Veterinary | | + | - | 3 | 1 | - | - | - | - | + | - | absent |  |  |  |  |  |  |  |
| DGF_0205 | | | DGF_0205_07 | | Veterinary | | + | - | 3 | 1 | - | - | - | - | + | - | absent | x | - | - | - | - | - | SRR3115502 |
| DGF_0205 | | | DGF_0205_08 | | Veterinary | | + | - | 3 | 1 | - | - | - | - | + | - | absent |  | NA | | | | | |
| DGF_0205 | | | DGF_0205_09 | | Veterinary | | + | - | 3 | 1 | - | - | - | - | + | - | absent |  |  |  |  |  |  |  |
| DGF_0205 | | | DGF_0205_10 | | Veterinary | | + | - | 3 | 1 | - | - | - | - | + | - | absent |  |  |  |  |  |  |  |
| DGF_0205 | | | DGF_0205_11 | | Veterinary | | + | - | 3 | 1 | - | - | - | - | + | - | absent | x | - | - | - | - | - | SRR3115503 |
| DGF_0214 | | | DGF_0214_01 | | Veterinary | | + | - | 15 | 1 | - | - | - | - | + | - | absent |  | NA | | | | | |
| DGF_0214 | | | DGF_0214_03 | | Veterinary | | + | - | 15 | 1 | - | - | - | - | + | - | absent |  |  |  |  |  |  |  |
| DGF_0214 | | | DGF_0214_04 | | Veterinary | | + | - | 15 | 1 | - | - | - | - | + | - | absent |  |  |  |  |  |  |  |
| DGF_0214 | | | DGF_0214_05 | | Veterinary | | + | - | 15 | 1 | - | - | - | - | + | - | absent |  |  |  |  |  |  |  |
| DGF_0214 | | | DGF_0214_07 | | Veterinary | | + | - | 15 | 1 | - | - | - | - | + | - | absent |  |  |  |  |  |  |  |
| DGF_0214 | | | DGF_0214_08 | | Veterinary | | + | - | 15 | 1 | - | - | - | - | + | - | absent |  |  |  |  |  |  |  |
| DGF_0214 | | | DGF_0214_09 | | Veterinary | | + | - | 15 | 1 | - | - | - | - | + | - | absent |  |  |  |  |  |  |  |
| DGF_0214 | | | DGF_0214_10 | | Veterinary | | + | - | 15 | 1 | - | - | - | - | + | - | absent |  |  |  |  |  |  |  |
| DGF_0214 | | | DGF_0214_11 | | Veterinary | | + | - | 15 | 1 | - | - | - | - | + | - | absent | x | - | + | - | - | - | SRR3115504 |
| DGF_0214 | | | DGF_0214_12 | | Veterinary | | + | - | 15 | 1 | - | - | - | - | + | - | absent |  | NA | | | | | |
| DGF_0214 | | | DGF_0214_13 | | Veterinary | | + | - | 15 | 1 | - | - | - | - | + | - | absent |  |  |  |  |  |  |  |
| DGF_0217 | | | DGF_0217_01 | | Veterinary | | + | - | 15 | 1 | - | - | - | - | + | - | absent | x | - | - | - | - | - | SRR3115505 |
| DGF_0217 | | | DGF_0217_02 | | Veterinary | | + | + | 42 | 1 | + | 3 | 3 | WT | - | - | A^+^B^+^C^WT^*cdtB*^-^ | x | - | - | - | - | - | SRR3115507 |
| DGF_0217 | | | DGF_0217_03 | | Veterinary | | + | - | 31 | 1 | - | - | - | - | + | - | absent | x | - | - | - | - | - | SRR3115506 |
| DGF_0217 | | | DGF_0217_04 | | Veterinary | | + | + | 42 | 1 | + | 3 | 3 | WT | - | - | A^+^B^+^C^WT^*cdtB*^-^ |  | NA | | | | | |
| DGF_0217 | | | DGF_0217_05 | | Veterinary | | + | - | 15 | 1 | - | - | - | - | + | - | absent |  |  |  |  |  |  |  |
| DGF_0217 | | | DGF_0217_06 | | Veterinary | | + | - | 31 | 1 | - | - | - | - | + | - | absent |  |  |  |  |  |  |  |
| DGF_0217 | | | DGF_0217_07 | | Veterinary | | + | - | 31 | 1 | - | - | - | - | + | - | absent |  |  |  |  |  |  |  |
| DGF_0217 | | | DGF_0217_08 | | Veterinary | | + | + | 42 | 1 | + | 3 | 3 | WT | - | - | A^+^B^+^C^WT^*cdtB*^-^ |  |  |  |  |  |  |  |
|  |  | |  | |  |  |  |  |  |  |  |  |  |  |  |  |  |  |  |  |  |  |  |  |

The 290 *Clostridium difficile* isolates represent 39 unique strains from 29 positive fecal samples (Fig 2). We sequenced typed all 290 isolates plus five fecal enrichment extractions (FEEs) (Fig 2, Table S1 and S2). The remaining three *cdiff* PCR positive FEEs contained extremely low concentrations of *C. difficile* DNA that were below our minimum technical limit needed to sequence type. None of the ten samples collected at two dog parks were positive for *C. difficile*.

**Bold:** Culturing was unsuccessful, but MLST was successfully performed on FEE.

**C. difficile* gDNA concentration below our technical limit to MLST type. Confirmed as *C. difficile* positive at one or two toxin associated loci.

†4 of 7 MLST markers sequenced successfully confirming this sample as a *C. difficile* positive. Unable to assign ST or identify co-colonization.

‡*tcdC* fragment allele

§WT, wild type

¶NA, not available
