# Supplementary material for: More than 50% of Clostridium difficile Isolates from Pet Dogs in Flagstaff, USA, Carry Toxigenic Genotypes
Source: PLoS One. 2016 Oct 10;11(10):e0164504. doi: 10.1371/journal.pone.0164504 (PMC5056695; doi:10.1371/journal.pone.0164504)
Supplement: S2 Table — (DOCX) [file pone.0164504.s002.docx]

**Supplemental Files: Domestic Canines are a Potential Source of Community Acquired *Clostridium difficile* Infections in Humans**

**Table S2. Summarized Table S1**

| Sample ID | Source | Isolates *n*= | MLST | | *Cdiff* TaqMan® | *tcdB* TaqMan® | Toxin genes | | | | | | | WGS | Resistance determinants | | | | | |
| --- | --- | --- | --- | --- | --- | --- | --- | --- | --- | --- | --- | --- | --- | --- | --- | --- | --- | --- | --- | --- |
|  |  |  | ST | Clade |  |  | *tcdA* | *tcdB* | *tcdC*^‡^ | *tcdC*Δ | *cdd1*/*cdu1* | *cdtB* toxin | Toxin genotype |  | *tet(M)* | | *erm(B)* | *gyrA* | *gyrB* | *rpoB* |
| Cdiff-ATCC | Positive Control | 2 | 1 | 2 | + | + | + | 8 | 1 | Δ1:Δ18 | - | + | A^+^B^+^C^Δ1Δ18^*cdtB*^+^ | 1 | - | | - | Thr-82→Ile | - | - |
| DGF_0001 | Systematic | 2 | 42 | 1 | + | + | + | 3 | 3 | WT | - | - | A^+^B^+^C^WT^*cdtB*^-^ | 1 | - | | - | - | - | - |
| DGF_0006 | Systematic | 1 | 15 | 1 | + | - | - | - | - | - | + | - | absent | 1 | - | | - | - | - | - |
| DGF_0006 | Systematic | 10 | 8 | 1 | + | + | + | 3 | 3 | WT | - | - | A^+^B^+^C^WT^*cdtB*^-^ | 2 | - | | - | - | - | - |
| DGF_0011 | Systematic | 10 | 3 | 1 | + | - | - | - | - | - | + | - | absent | 5 | - | | - | - | - | - |
| DGF_0027 | Systematic | 10 | 42 | 1 | + | + | + | 3 | 3 | WT | - | - | A^+^B^+^C^WT^*cdtB*^-^ | 1 | - | | - | - | - | - |
| DGF_0034 | Systematic | 7 | 3 | 1 | + | - | - | - | - | - | + | - | absent | 1 | - | | - | - | - | - |
| DGF_0036 | Systematic | 11 | 15 | 1 | + | - | - | - | - | - | + | - | absent | 1 | - | | - | - | - | - |
| DGF_0036 | Systematic | 4 | 42 | 1 | + | + | + | 3 | 3 | WT | - | - | A^+^B^+^C^WT^*cdtB*^-^ | 2 | - | | - | - | - | - |
| DGF_0040 | Systematic | 10 | 15 | 1 | + | - | - | - | - | - | + | - | absent | 1 | - | | - | - | - | - |
| DGF_0048 | Systematic | 12 | 42 | 1 | + | + | + | 3 | 3 | WT | - | - | A^+^B^+^C^WT^*cdtB*^-^ | 6 | - | | - | - | - | - |
| DGF_0048 | Systematic | 1 | 6 | 1 | + | + | + | 3 | 2 | WT | - | - | A^+^B^+^C^WT^*cdtB*^-^ | 1 | - | | - | - | - | - |
| DGF_0050 | Systematic | 17 | 42 | 1 | + | + | + | 3 | 3 | WT | - | - | A^+^B^+^C^WT^*cdtB*^-^ | 1 | - | | - | - | - | - |
| DGF_0059 | Systematic | 10 | 3 | 1 | + | - | - | - | - | - | + | - | absent | 1 | - | | - | - | - | - |
| DGF_0062 | Systematic | 12 | 28 | 1 | + | + | + | 3 | 7 | Δ18 | - | - | A^+^B^+^C^Δ18^*cdtB*^-^ | 2 | - | | - | - | - | - |
| DGF_0062 | Systematic | 5 | 15 | 1 | + | - | - | - | - | - | + | - | absent | 1 | - | | - | - | - | - |
| DGF_0063 | Systematic | 10 | 29 | 1 | + | - | - | - | - | - | + | - | absent | 1 | - | | - | - | - | - |
| **DGF_0065*** | Systematic | 0 | NA | NA | + | + | + | NA | 31 | WT | - | - | A^+^B^+^C^WT^*cdtB*^-^ | - | NA | | | | | |
| **DGF_0088†** | Systematic | 0 | NA | NA | + | + | + | 3 | 31 | WT | - | - | A^+^B^+^C^WT^*cdtB*^-^ | - |  |  |  |  |  |  |
| DGF_0092 | Systematic | 10 | 2 | 1 | + | + | + | 3 | 4 | WT | - | - | A^+^B^+^C^WT^*cdtB*^-^ | 1 | - | | - | - | - | - |
| DGF_0103 | Systematic | 8 | 2 | 1 | + | + | + | 3 | 4 | WT | - | - | A^+^B^+^C^WT^*cdtB*^-^ | 1 | - | | - | - | - | - |
| **DGF_0112** | Systematic | 0 | 42 | 1 | + | + | + | 3 | 3 | WT | - | - | A^+^B^+^C^WT^*cdtB*^-^ | - | NA | | | | | |
| DGF_0113 | Systematic | 4 | 15 | 1 | + | - | - | - | - | - | + | - | absent | 2 | - | | - | - | - | - |
| DGF_0113 | Systematic | 3 | 2 | 1 | + | + | + | 3 | 4 | WT | - | - | A^+^B^+^C^WT^*cdtB*^-^ | 1 | - | | - | - | - | - |
| DGF_0113 | Systematic | 3 | 10 | 1 | + | + | + | 3 | 7 | Δ18 | - | - | A^+^B^+^C^Δ18^*cdtB*^-^ | 1 | - | | - | - | - | - |
| DGF_0120 | Systematic | 10 | 3 | 1 | + | - | - | - | - | - | + | - | absent | 2 | - | | - | - | - | - |
| DGF_0132 | Systematic | 8 | 110 | 1 | + | + | + | 3 | 31 | WT | - | - | A^+^B^+^C^WT^*cdtB*^-^ | 1 | - | | - | - | - | - |
| DGF_0134 | Systematic | 14 | 2 | 1 | + | + | + | 3 | 4 | WT | - | - | A^+^B^+^C^WT^*cdtB*^-^ | 1 | - | | - | - | - | - |
| DGF_0134 | Systematic | 1 | 15 | 1 | + | - | - | - | - | - | + | - | absent | 1 | - | | + | - | - | - |
| **DGF_0148** | Systematic | 0 | 3 | 1 | + | - | - | - | - | - | + | - | absent | - | NA | | | | | |
| DGF_0153 | Systematic | 10 | 3 | 1 | + | - | - | - | - | - | + | - | absent | 1 | - | | - | - | - | - |
| DGF_0156 | Systematic | 2 | 42 | 1 | + | + | + | 3 | 3 | WT | - | - | A^+^B^+^C^WT^*cdtB*^-^ | 2 | - | | - | - | - | - |
| **DGF_0157*** | Systematic | 0 | NA | NA | + | - | - | - | - | - | + | - | absent | - | NA | | | | | |
| **DGF_0160** | Systematic | 0 | 15 | 1 | + | - | - | - | - | - | + | - | absent | - |  |  |  |  |  |  |
| **DGF_0163** | Systematic | 0 | 2 | 1 | + | + | + | 3 | 4 | WT | - | - | A^+^B^+^C^WT^*cdtB*^-^ | - |  |  |  |  |  |  |
| DGF_0172 | Systematic | 9 | 15 | 1 | + | - | - | - | - | - | + | - | absent | 1 | - | | - | - | - | - |
| DGF_0173 | Systematic | 10 | 15 | 1 | + | - | - | - | - | - | + | - | absent | 1 | - | | - | - | - | - |
| **DGF_0177** | Systematic | 0 | 42 | 1 | + | + | + | 3 | 3 | WT | - | - | A^+^B^+^C^WT^*cdtB*^-^ | - | NA | | | | | |
| DGF_0179 | Systematic | 1 | 2 | 1 | + | + | + | 3 | 4 | WT | - | - | A^+^B^+^C^WT^*cdtB*^-^ | - |  |  |  |  |  |  |
| DGF_0196 | Systematic | 9 | 2 | 1 | + | + | + | 3 | 4 | WT | - | - | A^+^B^+^C^WT^*cdtB*^-^ | 1 | - | - | | Thr-82→Ile | - | - |
| DGF_0196 | Systematic | 10 | 42 | 1 | + | + | + | 1 | 3 | WT | - | - | A^+^B^+^C^WT^*cdtB*^-^ | 1 | - | - | | - | - | - |
| DGF_0199 | Systematic | 6 | 3 | 1 | + | - | - | - | - | - | + | - | absent | 1 | - | - | | - | - | - |
| DGF_0201 | Veterinary | 10 | 26 | 1 | + | - | - | - | - | - | + | - | absent | 1 | + | + | | - | - | - |
| DGF_0205 | Veterinary | 11 | 3 | 1 | + | - | - | - | - | - | + | - | absent | 2 | - | - | | - | - | - |
| DGF_0214 | Veterinary | 11 | 15 | 1 | + | - | - | - | - | - | + | - | absent | 1 | - | + | | - | - | - |
| DGF_0217 | Veterinary | 2 | 15 | 1 | + | - | - | - | - | - | + | - | absent | 1 | - | - | | - | - | - |
| DGF_0217 | Veterinary | 3 | 31 | 1 | + | - | - | - | - | - | + | - | absent | 1 | - | - | | - | - | - |
| DGF_0217 | Veterinary | 3 | 42 | 1 | + | + | + | 3 | 3 | WT | - | - | A^+^B^+^C^WT^*cdtB*^-^ | 1 | - | - | | - | - | - |

**Bold:** Culturing was unsuccessful, but MLST was successfully performed on fecal enrichment extraction (FEE).

**C. difficile* gDNA concentration below our technical limit to MLST type. Confirmed as *C. difficile* positive at one or two toxin associated loci.

†4 of 7 MLST markers sequenced successfully confirming this sample as a *C. difficile* positive. Unable to assign ST or identify co-colonization.

‡*tcdC* fragment allele

§WT, wild type

¶NA, not available
